# Perfectly matched 20-nucleotide guide RNA sequences enable robust genome editing using high-fidelity SpCas9 nucleases

**DOI:** 10.1186/s13059-017-1325-9

**Published:** 2017-10-11

**Authors:** Dingbo Zhang, Huawei Zhang, Tingdong Li, Kunling Chen, Jin-Long Qiu, Caixia Gao

**Affiliations:** 1 State Key Laboratory of Plant Cell and Chromosome Engineering, and Center for Genome Editing, Institute of Genetics and Developmental Biology, Chinese Academy of Sciences, Beijing, China; 2 University of Chinese Academy of Sciences, Beijing, China; 3 State Key Laboratory of Plant Genomics, Institute of Microbiology, Chinese Academy of Sciences, Beijing, China

## Abstract

High-fidelity SpCas9 variants (eSpCas9 and SpCas9-HF1) have been engineered to reduce off-target effects. We found that changes in guide RNA length induced significant reductions in the editing activities of SpCas9 variants in plant cells. Single guide RNAs harboring precise, perfectly matched 20-nucleotide guide sequences are necessary for high on-target editing activities of eSpCas9 and SpCas9-HF1. Precise 20-nucleotide guide sequences derived from tRNA–sgRNA precursors enable robust on-target editing by these variants with enhanced specificity. Our work reveals an effective way of enhancing the use of the high-fidelity SpCas9 nucleases for efficient and precise genome engineering.

**Electronic supplementary material:**

The online version of this article (doi:10.1186/s13059-017-1325-9) contains supplementary material, which is available to authorized users.

## Background

The CRISPR-Cas9 system recognizes genomic sites via Watson-Crick base pairing by virtue of 20-nucleotide (nt) guide sequences in the guide RNAs (gRNAs) that direct Cas9 for targeted cleavage [1–4]. The CRISPR-Cas9 methodology is revolutionizing genome engineering and genetic therapy [5, 6]. However, Cas9 can also target DNA sequences that harbor one or multiple mismatches with gRNAs. Off-target effects are a serious concern in CRISPR-Cas9-mediated editing [7–9] and substantial efforts are consequently being made to minimize these [10–17]. Recently, three high-fidelity *Streptococcus pyogenes* Cas9 (SpCas9) variants, eSpCas9(1.0), eSpCas9(1.1), and SpCas9-HF1, were rationally engineered by amino acid substitutions to reduce non-specific interactions with its target DNA [18, 19]. While reducing non-specific DNA recognition, both eSpCas9 and SpCas9-HF1 maintain the efficacy of on-target cleavage. Nonetheless, a mismatched G at the 5′ end and truncation of the single-guide RNA (sgRNA) were noticed to reduce the nuclease activity of SpCas9-HF1 [19]. Therefore, it is important to identify the characteristics of sgRNAs that optimize the nuclease activities of these high-fidelity SpCas9 variants.

Here, we evaluated the efficiency and specificity of eSpCas9 and SpCas9-HF1 in plant genome editing processes, and found that precise perfectly matched 20-nt guide sequences could ensure the high efficiencies of these SpCas9 variants and maintain their high fidelity.

## Results

**Fig. 1:**
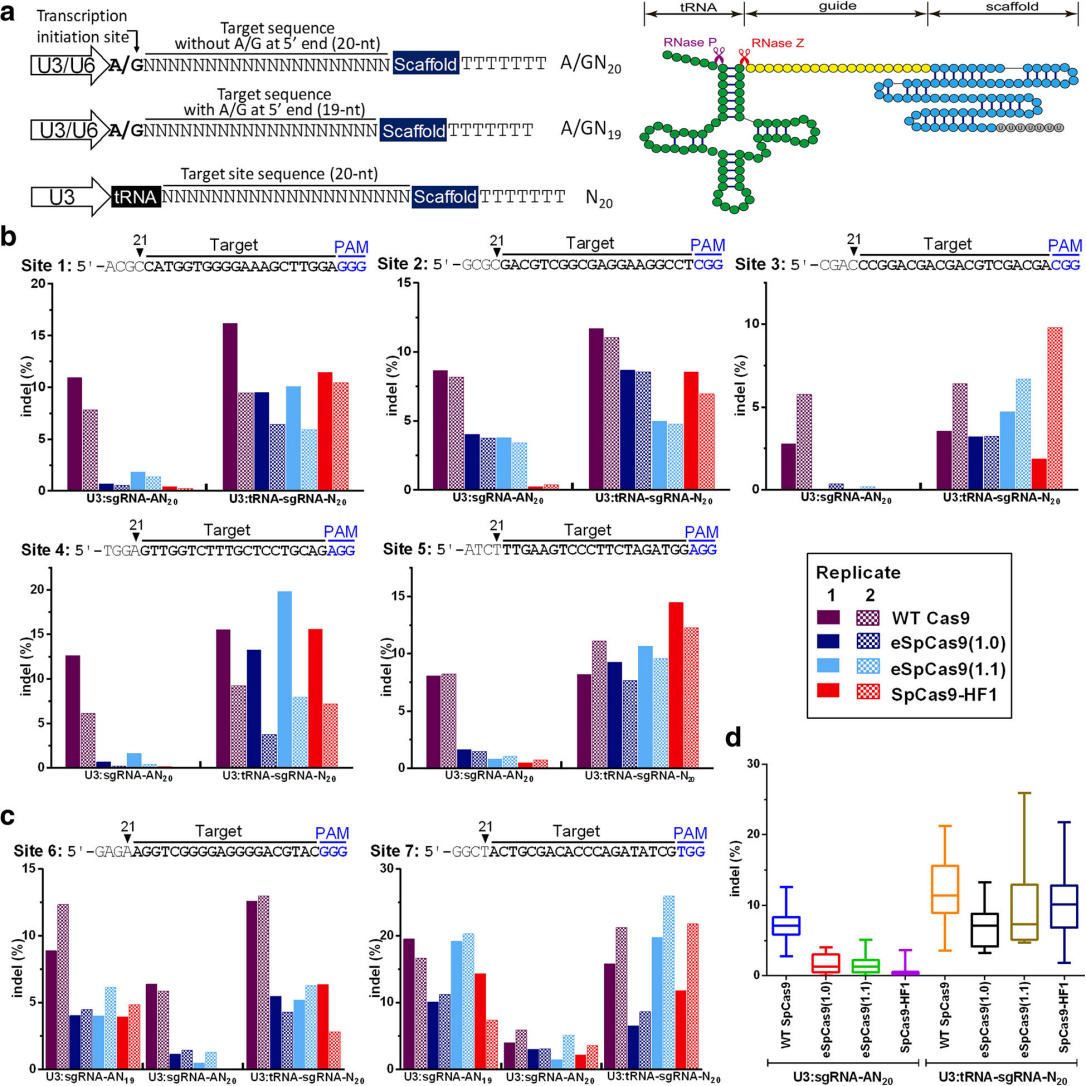
Analysis of the on-target activities of wild-type (WT) SpCas9 and three high-fidelity SpCas9 variants for seven genomic sites using different sgRNAs. **a** sgRNA constructs used and tRNA-mediated sgRNA processing. For the U3/U6 promoter, the transcription initiation site starts with A/G, so the transcribed sgRNA carries an A/G at the 5′ end. The sgRNAs are precisely processed from tRNA–sgRNA precursors. Endogenous RNase P and RNase Z cleave the transcripts and release mature sgRNAs. **b** Comparison of the on-target activities of WT SpCas9 and three variants at five genomic sites (sites 1–5) without A at their 5′ ends using U3:sgRNA-AN_20_ or U3:tRNA-sgRNA-N_20_. **c** Comparison of the corresponding on-target activities at two genomic sites (sites 6 and 7) with A at their 5′ ends using U3:sgRNA-AN_19_, U3:sgRNA-AN_20_, or U3:tRNA-sgRNA-N_20_. Two independent replicates were performed. *Solid filled columns* indicate replicate 1 and *pattern filled columns* indicate replicate 2. **d** Summary of the on-target activities of three SpCas9 variants using U3:sgRNA-AN20 or U3:tRNA-sgRNA-N20 compared to WT SpCas9 in **b**, **c**

We first tested the activities of the three SpCas9 variants on seven different genomic sites in rice protoplasts (Additional file 1: Table S1). The coding sequences of eSpCas9(1.0), eSpCas9(1.1), and SpCas9-HF1 were cloned into pJIT163 under the control of the maize *Ubiquitin 1* promoter (Additional file 2: Sequences). These constructs were independently transformed into rice protoplasts together with each of the seven OsU3:sgRNA constructs [20]. Because transcripts made under the control of the eukaryotic U3 and U6 promoters generally start with an adenine (A) or guanine (G), the 5′ ends of the mature sgRNAs carry an A or G which may not match the target sequences. Therefore, for those target sites with an A or G at their 5′ ends, we designed the guide sequences of sgRNAs with a 19-nt (N_19_) sequence beside this A or G, followed by the PAM (protospacer adjacent motif), so that the sequences of the mature sgRNA would be AN_19_ or GN_19_. We also designed 20-nt (N_20_) guide sequences so that the mature sgRNA guide sequence would be AN_20_ or GN_20_ (Fig. 1a; Additional file 1: Figure S1). Hence, for sites 1 to 5 in Fig. 1, which do not start with an A, the guide sequences were 21-nt in length (AN_20_) with an additional matched or non-matched A at their 5′ ends (Fig. 1b). For sites 6 and 7, which start with an A, the guide sequences were 20-nt (AN_19_) in length and were perfectly complementary to the target sites (Fig. 1c).

Two days after protoplast transfection, deep amplicon sequencing was performed to determine the frequencies of on-target indels (insertions and deletions). As shown in Fig. 1 and Additional file 1: Table S2, wild-type (WT) SpCas9 exhibited high on-target activity (3–20%) for all seven sites. Surprisingly, the on-target activities of the three SpCas9 variants were considerably lower. For sites 1 to 5, they were almost negligible for SpCas9-HF1, and for eSpCas9(1.0) and eSpCas9(1.1) they were still much lower than for WT SpCas9 (Fig. 1b). For sites 6 and 7, however, the three variants exhibited 40% or more of the on-target activities observed with WT SpCas9 (Fig. 1c). These data suggest that an extra adenine at the 5′ end of an sgRNA-AN_20_ sequence reduced the on-target activities of the three SpCas9 variants, whether or not the extra A matched (site 4) or did not match (sites 1, 2, 3, 5) the target DNA sequence (Fig. 1d). To test this idea, we introduced an extra adenine at the 5′ ends of the sgRNAs targeting sites 6 and 7 (Fig. 1c). As expected, the SpCas9 variants, when used with a sgRNA-AN_20_ sequence, had significantly reduced on-target cleavage activities, even though in the case of site 6, at least, the AN_20_ sequence was precisely complementary to the genomic sequence (Fig. 1c).

Like the U3 promoter, the U6 promoter is widely used to drive sgRNA expression [21]. We investigated whether the on-target activities of eSpCas9(1.0), eSpCas9(1.1), and SpCas9-HF1 were also compromised by the 21-nt guide sequence of sgRNAs transcribed by the U6 promoter. For this purpose we selected site 4, which has a G at its 5′ end (Fig. 1b), and used two sgRNA constructs driven by the U6 promoter of wheat, U6:sgRNA-GN_19_ (producing a precisely matching 20-nt sgRNA) and U6:sgRNA-GN_20_ (producing a 21-nt sgRNA due to an extra G at the 5′ end). The three SpCas9 variants induced similar numbers of on-target changes to WT SpCas9 with U6:sgRNA-GN_19_ but much reduced numbers when U6:sgRNA-GN_20_ was used (Additional file 1: Figure S2), further supporting the idea that an extra nucleotide at the 5′ end of the guide sequence compromises the on-target activities of the three variants. Together, these data confirm that the three SpCas9 variants have a stringent requirement for sgRNAs of precisely 20 nt for efficient on-target editing.

The above data encouraged us to seek an efficient way to generate sgRNAs of the exact length needed for the SpCas9 variants, in order to enhance their utility. Previous studies have shown that the endogenous tRNA-processing system can be harnessed to produce sgRNAs with precisely controlled guide sequences [22]. Once transcribed, the tRNA–sgRNA precursor can be efficiently processed by the cellular enzymes RNase P and RNase Z at predefined sites (Fig. 1a), producing 20-nt guide sequences completely complementary to the target sites. We therefore generated U3:tRNA–sgRNA constructs for sites 1 to 7 (Fig. 1a) and assessed their on-target cleavage activities when used with eSpCas9(1.0), eSpCas9(1.1), and SpCas9-HF1 (Fig. 1b–d). As expected, the variant SpCas9 enzymes and WT SpCas9 had similar nuclease activities with the sgRNAs produced from the tRNA–sgRNA precursors (Fig. 1b–d). For sites 1–5, the on-target activities of these variants were much higher than with the 21-nt guide sequences of sgRNAs generated without the tRNA–sgRNA expression system (Fig. 1b). The improvement was generally much greater for eSpCas9(1.1) and SpCas9-HF1 than for eSpCas9(1.0), and for sites 1, 3, 4, and 5, the on-target activities of eSpCas9(1.1) and SpCas9-HF1 were close to, or even higher than, those of WT SpCas9 (Fig. 1b). The on-target activity of WT SpCas9 was also increased, but the extent of the increase was much less (Fig. 1b). For sites 6 and 7, the on-target activities of the three variants with the sgRNAs produced from U3:tRNA–sgRNA constructs were comparable to those obtained with the sgRNAs-AN_19_ (Fig. 1c). These results further demonstrate that a perfectly matched 20-nt guide sequence is needed for optimal nuclease activities of eSpCas9(1.0), eSpCas9(1.1), and SpCas9-HF1. We propose that synthetic genes with a tRNA–sgRNA architecture provide a general strategy for producing sgRNAs with perfectly matched 20-nt sequences for use with the high-fidelity SpCas9 variants.

**Fig. 2:**
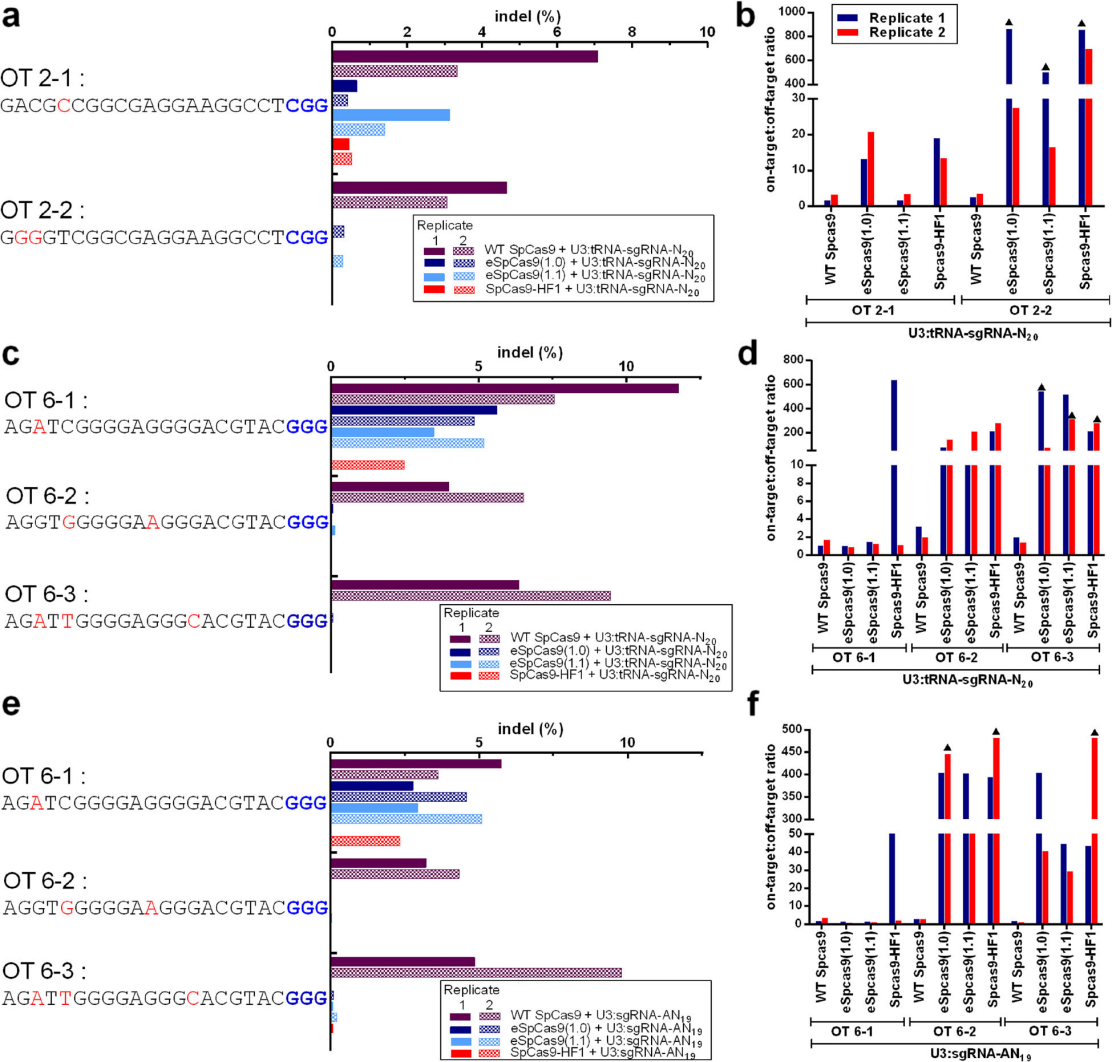
Comparisons of the off-target editing activities of WT SpCas9 and three high-fidelity SpCas9 variants at five off-target sites with sgRNAs produced from U3:tRNA–sgRNA constructs and U3:sgRNA constructs. **a**, **c**, **e** Activities of WT SpCas9, eSpCas9(1.0), eSpCas9(1.1), and SpCas9-HF1 for the two off-targets of site 2 (OT2-1 and OT2-2) and the three off-targets of site 6 (OT6-1, OT6-2, and OT6-3) using sgRNAs produced from U3:tRNA–sgRNA constructs (**a** and **c**) and for the three off-targets of site 6 with sgRNAs produced from U3:sgRNA-AN_19_ constructs (**e**). The off-targets had one (OT2-1 and OT6-1), two (OT2-2 and OT6-2), and three (OT6-3) mismatches (highlighted in *red*) to sites 2 and 6, respectively. The PAM is shown in *blue*. The percentage of indels was used to measure off-target editing activity. Two independent replicates were performed. *Solid filled columns* indicate replicate 1 and *pattern filled columns* indicate replicate 2. **b**, **d**, **f** Specificities of WT SpCas9, eSpCas9(1.0), eSpCas9(1.1), and SpCas9-HF1 represented as on-target:off-target indel frequency ratios. On-target:off-target ratios were calculated by dividing the on-target indel frequency by the off-target frequency. When off-target activity was undetectable (the threshold of detection was 0.01% of sequencing reads), we set the off-target efficiency to the threshold of detection (0.01%) and these cases are denoted by a *triangle*

**Fig. 3:**
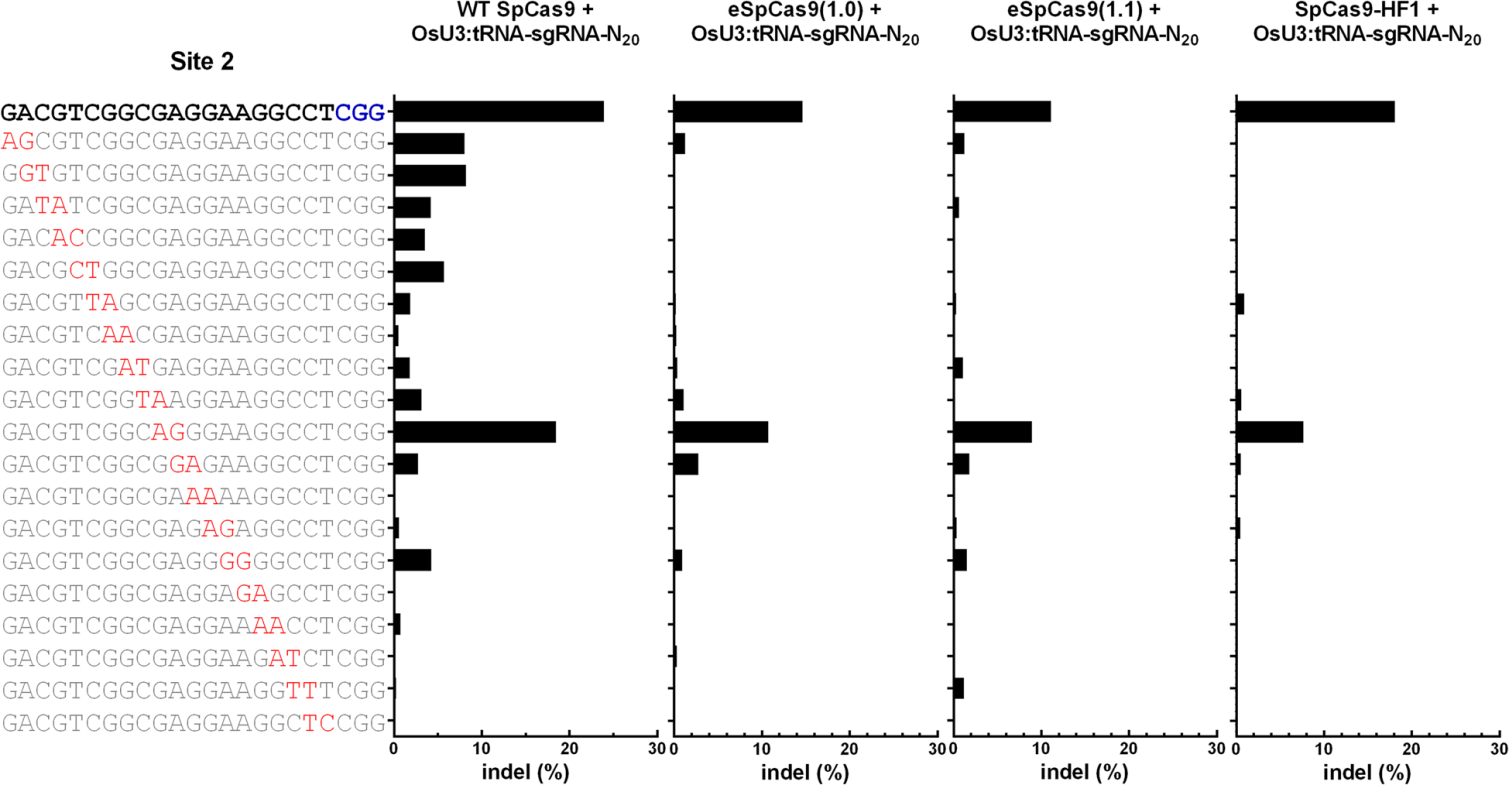
Comparisons of the specificities of WT SpCas9 and eSpCas9(1.0), eSpCas9(1.1), and SpCas9-HF1 with tRNA–sgRNA constructs. Off-target effects of WT SpCas9 and variants eSpCas9(1.0), eSpCas9(1.1), and SpCas9-HF1 with guide sequences containing pairs of mismatches at successive positions. The WT guide sequence is highlighted in *bold* with the PAM highlighted in *blue*. The WT guide sequence was systematically mutated by introducing pairs of mismatches at successive positions. A total of 20 sgRNAs (one WT guide sequence and 19 mutated guide sequences) were produced using U3:tRNA–sgRNA-N20 constructs. Each sgRNA was tested in combination with the four SpCas9 nucleases, and the percentage of indels was used to measure editing activity. The mismatch sites are highlighted in *red*

It has been shown that the amounts of U3 promoter-derived sgRNA transcripts are greater with synthetic tRNA–sgRNA genes compared to those from sgRNA alone [22]. This phenomenon was also apparent in an analysis using sites 2 and 6 as representatives (Additional file 1: Figure S3). Although the increased sgRNA abundance may be beneficial for the on-target activities of the three SpCas9 variants, it seemed possible that it would also stimulate off-target activities. This prompted us to examine the off-target editing activities of eSpCas9(1.0), eSpCas9(1.1), and SpCas9-HF1 with the sgRNAs produced from U3:tRNA–sgRNA constructs. Two off-targets of site 2 (OT2-1 and OT2-2) and three off-targets of site 6 (OT6-1, OT6-2, and OT6-3) were used in these experiments (Fig. 2). In general, the three SpCas9 variants had substantially less off-target activity than WT SpCas9, and this was particularly evident for OT2-2, OT6-2, and OT6-3 (Fig. 2a–d). SpCas9-HF1 consistently exhibited the lowest off-target activities at the five examined sites (Fig. 2a–d). The amounts of off-target activity obtained with U3:tRNA–sgRNA-N_20_ and U3:sgRNA-AN_19_ were similar (Fig. 2c–f). In addition, the on-target:off-target indel frequency ratios for the three SpCas9 variants were, on average, 273-fold higher than those for WT SpCas9 (Fig. 2b, d, f; Additional file 1: Table S2). Thus, the SpCas9 variants retained their high degree of specificity when used with guides produced from tRNA–sgRNA constructs. The off-target activities of the three SpCas9 variants were also investigated by systematically mutating the guide sequence of site 2 and checking the effects on editing of site 2 (Fig. 3). Pairs of mismatches were introduced at successive positions along the guide sequence and the resulting mutants were each fused with tRNA, the tRNA–sgRNA being transcribed under the U3 promoter. Compared with WT SpCas9, the three SpCas9 variants consistently induced much lower levels of indels with the mutant sgRNAs (Fig. 3). Collectively, the results of this series of experiments indicate that the three variant enzymes coupled with 20-nt sgRNA using tRNA–sgRNA fusions still possess high fidelity.

## Discussion

The specificity of Cas9 is of fundamental importance for its application. Double strand breaks (DSBs) induced at off-target sites can trigger cellular damage, and the repair of DSBs at off-target sites by non-homologous end joining (NHEJ) or homology-directed repair (HDR) may also cause unwanted mutations. Several strategies have been developed to improve the specificity of SpCas9, such as using computational tools to design the guide RNAs [23, 24], improving off-target detection methods [25–29], protein engineering [10–14, 16–19], modifying the guide RNAs [14, 15], and delivering purified Cas9 ribonucleoproteins (RNPs) into cells [30, 31]. The high-fidelity SpCas9 variants eSpCas9 and SpCas9-HF1, which were obtained by protein engineering, display extremely high specificity in mammalian cells.

In this study, we observed that both eSpCas9 and SpCas9-HF1 had stricter requirements concerning sgRNA guide sequences than WT SpCas9. When additional matched or mismatched residues were added to the 5′ end of N_20_ sgRNAs, the on-target activities of the high-fidelity SpCas9 variants decreased dramatically. However, when we used a tRNA–sgRNA expression system to produce precise N_20_ guide RNAs, the on-target activity of the SpCas9 variants was restored and could even be higher than that of WT SpCas9. In agreement with previous research, we found that eSpCas9 and SpCas9-HF1 exhibited high specificity and SpCas9-HF1 barely introduced any indels, especially at off-target sites with two or more mismatches. Since the tRNA–sgRNA architecture has been demonstrated to work well in *Drosophila* [32], we believe that our strategy can also work in beneficial ways in mammalian cells, and this will be tested in our future work.

The feasibility of our strategy has been proven in rice protoplasts. Next, we will use this strategy to generate mutant plants and compare the specificities of WT SpCas9 and the high-fidelity SpCas9 variants. An unbiased approach over the whole genome assessing off-target effects will be required to do this. In addition, knowledge of their crystal structures and enzyme kinetics might be helpful. Recent studies indicate that the type V CRISPR system nuclease Cpf1 is a highly specific RNA-guided endonuclease [33, 34]. It will be of interest to compare the genome-wide specificities of Cpf1 and eSpCas9/SpCas9-HF1. It has been reported that the high-fidelity base editor HF-BE3 [35], in which SpCas9-HF1 (containing the substitution D10A) is fused to cytidine deaminase and UGI, has greatly enhanced specificity. Hence, we believe that a fusion of eSpCas9 with cytidine deaminase and UGI could also have increased specificity.

## Conclusions

We have shown that producing sgRNAs intracellularly from tRNA–sgRNA transcripts increases the on-target activities of the high-specificity SpCas9 variants without sacrificing their high specificity. This approach should enhance the utility of these variant enzymes for efficient and precise genome engineering. We also believe that other means of accurately generating sgRNAs, e.g., using self-cleaving HDV and HH ribozymes or the endoribonuclease Csy4 [36, 37], could improve the on-target editing activity of these spCas9 variants without sacrificing their high specificity.

## Methods

### Plasmids

pJIT163-Ubi-2XNLS-Cas9 is a plasmid used in our previous study [20]. Point mutations were introduced to the coding sequence of SpCas9 with the Fast MultiSite Mutagenesis System (TransGen Biotech, Beijing, China), resulting in alternative expression cassettes producing eSpCas9(1.0) (K810A, K1003A, R1060A), eSpCas9(1.1) (K848A, K1003A, R1060A), or SpCas9-HF1 (N497A, R661A, Q695A, Q926A). OsU3:sgRNA and TaU6:sgRNA constructs were made using our published protocol [38]. Following the previous report, a generic tRNA–sgRNA cassette containing two FokI restriction sites was constructed [22], which was then employed to prepare the various tRNA–sgRNA constructs used in this work. The oligonucleotide primers used in this work are listed in Additional file 1: Tables S1, S3, and S4.

### Protoplast transfection

Rice cultivar Nipponbare was used throughout this work. Preparation of protoplasts from 2-week-old seedlings and transformation of the resultant protoplasts with desirable plasmid constructs were conducted as reported previously [20]. The average transformation efficiency of protoplasts was higher than 50%. In each transformation, the appropriate Cas9 and sgRNA constructs (10 μg each) were mixed and co-delivered into the protoplasts via PEG-mediated transfection.

### DNA extraction

The transfected protoplasts were incubated at 23 °C. After 48 h of incubation, they were harvested for genomic DNA extraction using the DNA quick Plant System (TIANGEN BIOTECH, Beijing, China). The targeted site was amplified by specific primers, with amplicons purified using the EasyPure PCR Purification Kit (TransGen Biotech, Beijing, China), and quantified with a NanoDrop™ 2000 Spectrophotometer (Thermo Fisher Scientific, Waltham, MA, USA).

### Off-target detection

Off-target detection was based on predictions using the online tool CRISPR-P [39] and previous research data [40]. The off-target sites for sites 2 and 6 in the rice genome were identified and verified in this work.

### RNA extraction and quantitative RT-PCR analysis of sgRNA expression

Total RNA samples were prepared from transfected rice protoplasts using the TRIzol reagent (Life Technologies, Carlsbad, CA, USA). After treatment with RNase-free DNase I (Life Technologies, Carlsbad, CA, USA), the samples were stored at −80 °C until use. For quantitative RT-PCR, an aliquot of the total RNA (2 μg) was reverse transcribed into cDNA using oligo dT and sgRNA specificity primer (Additional file 1: Table S4) and M-MLV reverse transcriptase (Promega, Madison, WI, USA) following the manufacturer’s instruction. The qRT-PCR was then performed using SsoFast EvaGreen Supermix (Bio-Rad, Foster City, CA, USA) in a CFX 384 Touch Real-Time RCR Detection System (Bio-Rad, Foster City, CA, USA) to measure sgRNA expression level. The rice Ubiquitin gene (*LOC_Os02g06640*) was used as an internal control for quantitative RT-PCR [22].

### Deep amplicon sequencing

Specific primers were used to amplify the genomic regions flanking the CRISPR target site in the first round PCR. The resultant PCR products were subjected to a second round PCR, with forward and reverse barcodes added to the products. Primers are listed in Additional file 1: Table S5. Equal amounts of final PCR products were mixed and pooled for library construction. Then the libraries were sequenced commercially (Mega Genomics, Beijing, China) by paired-end read sequencing using the Illumina NextSeq 500 platform. The indels detected inside the target site were considered as evidence of mutagenesis [41, 42].

## Additional file

Additional file 1: Figure S1. Complete DNA sequences of the sgRNA expression constructs. **Figure S2.** Comparison of the on-target activities of WT SpCas9 and its variants. **Figure S3.** The relative expression levels of the sgRNAs produced from two types of sgRNA constructs. **Table S1.** Target sequences and oligos used to construct sgRNA expression vectors. **Table S2.** Indel frequencies revealed by deep amplicon sequencing. **Table S3.** Oligos used to construct vectors of sgRNA with mismatches. **Table S4.** PCR primers used in this study. **Table S5.** Second round PCR primers with barcodes for deep amplicon sequencing. (PDF 766 kb)
Additional file 2: Rice codon optimized DNA sequences of WT Cas9, eSpCas9(1.0), eSpCas9(1.1), and SpCas9-HF1. (PDF 296 kb)
