# Supplementary material for: Perfectly matched 20-nucleotide guide RNA sequences enable robust genome editing using high-fidelity SpCas9 nucleases
Source: Genome Biol. 2017 Oct 11;18:191. doi: 10.1186/s13059-017-1325-9 (PMC5637269; doi:10.1186/s13059-017-1325-9)
Supplement: Additional file 1: Figure S1. — Complete DNA sequences of the sgRNA expression constructs. Figure S2. Comparison of the on-target activities of WT SpCas9 and its variants. Figure S3. The relative expression levels of the sgRNAs produced from two types of sgRNA constructs. Table S1. Target sequences and oligos used to construct sgRNA expression vectors. Table S2. Indel frequencies revealed by deep amplicon sequencing. Table S3. Oligos used to construct vectors of sgRNA with mismatches. Table S4. PCR primers used in this study. Table S5. Second round PCR primers with barcodes for deep amplicon sequencing. (PDF 766 kb) [file 13059_2017_1325_MOESM1_ESM.pdf]

a

U3:sgRNA-N_{19}/N_{20}

AAGGAATCTTTAAACATACGAACAGATCACTTAAAGTTCTTCTGAAGCAACTTAAAGTTATCAGGCATGCATGGATCTTGGAGGAATCAGATGTGCAGTCAGGGACCATA
GCACAAGACAGGCGTCTTCTACTGGTGCTACCAGCAAATGCTGGAAGCCGGGAACACTGGGTACGTCGGAAACCACGTGATGTGAAGAAGTAAGATAAACTGTAGGAGAA
AAGCATTTCGTAGTGGGCCATGAAGCCTTTCAGGACATGTATTGCAGTATGGGCCGGCCCATTACGCAATTGGACGACAACAAAGACTAGTATTAGTACCACCTCGGCTA
TCCACATAGATCAAAGCTGATTTAAAAGAGTTGTGCAGATGATCCGTGGCA**N_{19}/N_{20}**GTTTTAGAGCTAGAAATAGCAAGTTAAAATAAGGCTAGTCCGTTATCAACTTGA
AAAAGTGGCACCGAGTCGGTGCTTTTTTT

b

U3:tRNA-sgRNA-N_{20}

AAGGAATCTTTAAACATACGAACAGATCACTTAAAGTTCTTCTGAAGCAACTTAAAGTTATCAGGCATGCATGGATCTTGGAGGAATCAGATGTGCAGTCAGGGACCATA
GCACAAGACAGGCGTCTTCTACTGGTGCTACCAGCAAATGCTGGAAGCCGGGAACACTGGGTACGTCGGAAACCACGTGATGTGAAGAAGTAAGATAAACTGTAGGAGAA
AAGCATTTCGTAGTGGGCCATGAAGCCTTTCAGGACATGTATTGCAGTATGGGCCGGCCCATTACGCAATTGGACGACAACAAAGACTAGTATTAGTACCACCTCGGCTA
TCCACATAGATCAAAGCTGATTTAAAAGAGTTGTGCAGATGATCCGTGGCAACAAAGCACCAGTGGTCTAGTGGTAGAATAGTACCCTGCCACGGTACAGACCCGGGTTC
GATTCCCGGCTGGTGCA**N_{20}**GTTTTAGAGCTAGAAATAGCAAGTTAAAATAAGGCTAGTCCGTTATCAACTTGAAAAAGTGGCACCGAGTCGGTGCTTTTTTT

c

U6:sgRNA-N_{19}/N_{20}

GACCAAGCCCGTTATTCTGACAGTTCTGGTGCTCAACACATTTATATTTATCAAGGAGCACATTGTTACTCACTGCTAGGAGGGAATCGAACTAGGAATATTGATCAGAG
GAACTACGAGAGAGCTGAAGATAACTGCCCTCTAGCTCTCACTGATCTGGGTCGCATAGTGAGATGCAGCCCACGTGAGTTCAGCAACGGTCTAGCGCTGGGCTTTTAGG
CCCGCATGATCGGGCTTTTGTCGGGTGGTCGACGTGTTCACGATTGGGGAGAGCAACGCAGCAGTTCCTCTTAGTTTAGTCCCACCTCGCCTGTCCAGCAGAGTTCTGAC
CGGTTTATAAACTCGCTTGCTGCATCAGACTTG**N_{19}/N_{20}**GTTTTAGAGCTAGAAATAGCAAGTTAAAATAAGGCTAGTCCGTTATCAACTTGAAAAAGTGGCACCGAGTCG
GTGCTTTTTTT

Figure S1. Complete DNA sequences of the sgRNA expression constructs, U3:sgRNA-N_{19}/N_{20} (a), U3:tRNA-sgRNA-N_{20} (b) and U6:sgRNA-N_{19}/N_{20} (c). The rice U3 promoter is highlighted in blue, and the wheat U6 promoter is highlighted in purple. The target sequences are indicated by N_{19}/N_{20} in bold and the sgRNA scaffolds are colored in red. The transcription initiation sites of rice U3 promoter and wheat U6 promoter are indicated in green background. The polymerase III terminators (poly T) are indicated in grey background.

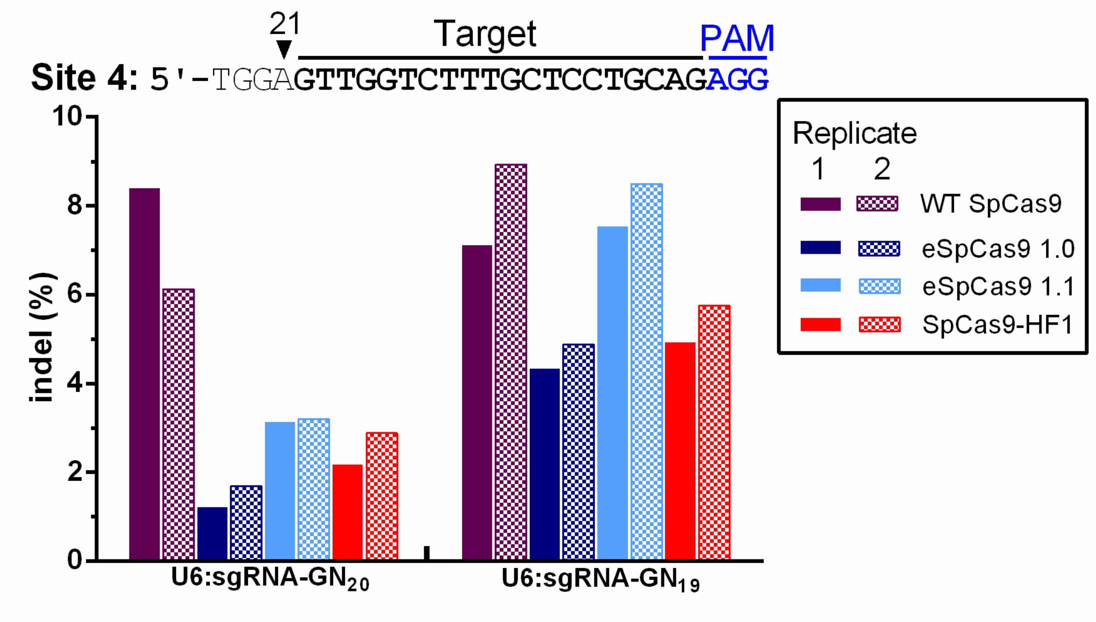

Figure S2. Comparison of the on-target activities of WT SpCas9 and variants eSpCas9(1.0), eSpCas9(1.1) and SpCas9-HF1 for Site 4 with G at its 5' end using U6:sgRNA-GN_{20} or U6:sgRNA-GN_{19}. Two independent replicates were performed. Solid filled columns indicate replicate 1 and pattern filled columns indicate replicate 2.

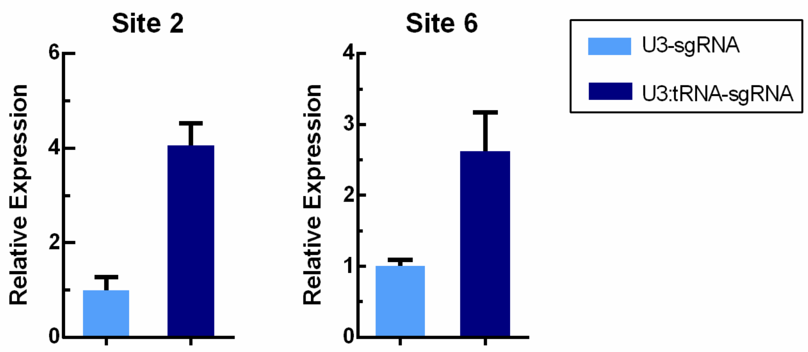

Figure S3. The relative expression levels of the sgRNAs produced from two types of sgRNA constructs, i.e., U3:sgRNA and U3:tRNA-sgRNA. Error bars are SDs (n = 4).

Table S1. Target sequences and oligos used to construct sgRNA expression vectors.

Target site and gene identifiers	Target sequence (5'-3')	Oligo (5'-3')	Application
Site 1: LOC_Os01g55540	CATGGTGGGGAAAGCTTGGA **GGG**	F:GGCACATGGTGGGGAAAGCTTGGA<br>R:AAACTCCAAGCTTTCCCCACCATG	constructing pU3-sgRNA
		F:TAGGTCTCCGGAAAGCTTGGAgttttagagctagaa<br>R:ATGGTCTCATTCCCCACCATGtgcaccagccgggaa	constructing pU3:tRNA-sgRNA
Site 2: LOC_Os02g54600	GACGTCGGCGAGGAAGGCCT **CGG**	F:GGCAGACGTCGGCGAGGAAGGCCT<br>R:AAACAGGCCTTCCTCGCCGACGTC	constructing pU3-sgRNA
		F:TAGGTCTCCCGAGGAAGGCCTgttttagagctagaa<br>R:ATGGTCTCACTCGCCGACGTCtgcaccagccgggaa	constructing pU3:tRNA-sgRNA
Site 3: LOC_Os02g54600	CCGGACGACGACGTCGACGA **CGG**	F:GGCACCGGACGACGACGTCGACGA<br>R:AAACTCGTCGACGTCGTCGTCCGG	constructing pU3-sgRNA
		F:TAGGTCTCCCGACGTCGACGAgttttagagctagaa<br>R:ATGGTCTCAGTCGTCGTCCGGtgcaccagccgggaa	constructing pU3:tRNA-sgRNA
Site 4: LOC_Os03g0857	GTTGGTCTTTGCTCCTGCAG **AGG**	F:GGCAGTTGGTCTTTGCTCCTGCAG<br>R:AAACCTGCAGGAGCAAAGACCAAC	constructing pU3-sgRNA
		F:TAGGTCTCCTTGCTCCTGCAGgttttagagctagaa<br>R:ATGGTCTCAGCAAAGACCAACtgcaccagccgggaa	constructing pU3:tRNA-sgRNA
		F:CTTGGTTGGTCTTTGCTCCTGCAG<br>R:AAACCTGCAGGAGCAAAGACCAAC	constructing pU6-sgRNA
		F:CTTGTTGGTCTTTGCTCCTGCAG	

		R:AAACCTGCAGGAGCAAAGACCAA	
Site 5: LOC_Os05g49140	TTGAAGTCCCTTCTAGATGG **AGG**	F:GGCATTGAAGTCCCTTCTAGATGG<br>R:AAACCCATCTAGAAGGGACTTCAA	constructing pU3-sgRNA
		F:TAGGTCTCCCCTTCTAGATGGgttttagagctagaa<br>R:ATGGTCTCAAAGGGACTTCAAtgcaccagccgggaa	constructing pU3:tRNA-sgRNA
Site 6: LOC_Os08g40170	AGGTCGGGGAGGGGACGTAC **GGG**	F:GGCAGGTCGGGGAGGGGACGTAC<br>R:AAACGTACGTCCCCTCCCCGACC	constructing pU3-sgRNA
		F:GGCAAGGTCGGGGAGGGGACGTAC<br>R:AAACGTACGTCCCCTCCCCGACCT	
		F:TAGGTCTCCGAGGGGACGTACgttttagagctagaa<br>R:ATGGTCTCACCTCCCCGACCTtgcaccagccgggaa	constructing pU3:tRNA-sgRNA
Site 7: LOC_Os11g17080	ACTGCGACACCCAGATATCG **TGG**	F:GGCACTGCGACACCCAGATATCG<br>R:AAACCGATATCTGGGTGTCGCAG	constructing pU3-sgRNA
		F:GGCAACTGCGACACCCAGATATCG<br>R:AAACCGATATCTGGGTGTCGCAGT	
		F:TAGGTCTCCACCCAGATATCGgttttagagctagaa<br>R:ATGGTCTCAGGGTGTCGCAGTtgcaccagccgggaa	constructing pU3:tRNA-sgRNA
OT2-1: LOC_Os04g52140	GACGCCGGCGAGGAAGGCCT **CGG**		
OT2-2: LOC_Os02g14110	GGGGTCGGCGAGGAAGGCCT **CGG**		
OT6-1: LOC_Os02g03060	AGATCGGGGAGGGGACGTAC **GGG**		
OT6-2:	AGGTGGGGGAAGGGACGTAC		

LOC_Os01g67160	**GGG**		
OT6-3: LOC_Os03g02680	AGATTGGGGAGGGCACGTAC **GGG**		

The PAM is shown in bold. The mismatch sites are highlighted in red.

Table S2. Indel frequencies revealed by deep amplicon sequencing.

Target site	sgRNA construction	SpCas9 variants	Experiment repeat	Total reads	Indels reads	Indel frequence (%)
Site 1	U3-sgRNA-AN_{20}	WT SpCas9	1	14280	1567	10.97%
			2	11845	934	7.89%
		eSpCas9(1.0)	1	5287	39	0.74%
			2	4773	28	0.59%
		eSpCas9(1.1)	1	14813	280	1.89%
			2	13080	184	1.41%
		SpCas9-HF1	1	28109	134	0.48%
			2	17151	53	0.31%
	U3:tRNA-sgRNA-N_{20}	WT SpCas9	1	11588	1882	16.24%
			2	12354	1178	9.54%
		eSpCas9(1.0)	1	21956	2105	9.59%
			2	12433	807	6.49%
		eSpCas9(1.1)	1	11237	1138	10.13%
			2	8402	503	5.99%
		SpCas9-HF1	1	20693	2383	11.52%
			2	9593	1007	10.50%
	Control		1	33125	19	0.06%
			2	14134	9	0.06%
Site 2	U3-sgRNA-AN_{20}	WT SpCas9	1	8838	779	8.81%
			2	4610	378	8.20%
		eSpCas9(1.0)	1	67837	2863	4.22%
			2	26345	1001	3.80%
		eSpCas9(1.1)	1	2762	109	3.95%
			2	1709	59	3.45%
		SpCas9-HF1	1	312131	1325	0.42%
			2	116074	452	0.39%
	U3:tRNA-sgRNA-N_{20}	WT SpCas9	1	61038	7243	11.87%
			2	25935	2869	11.06%
		eSpCas9(1.0)	1	124983	11033	8.83%
			2	52476	4495	8.57%
		eSpCas9(1.1)	1	297925	15335	5.15%
			2	110981	5351	4.82%
		SpCas9-HF1	1	6056	527	8.70%
			2	3170	222	7.00%
	Control		1	49177	83	0.17%
			2	22263	7	0.03%
Site 3	U3-sgRNA-AN_{20}	WT SpCas9	1	30341	864	2.85%
			2	26262	1531	5.83%
		eSpCas9(1.0)	1	14628	18	0.12%
			2	14259	61	0.43%
		eSpCas9(1.1)	1	30930	34	0.11%
			2	31782	85	0.27%
		SpCas9-HF1	1	41496	23	0.06%
			2	29830	30	0.10%
	U3:tRNA-sgRNA-N_{20}	WT SpCas9	1	27921	1014	3.63%
			2	27307	1762	6.45%
		eSpCas9(1.0)	1	40089	1318	3.29%
			2	30916	1018	3.29%
		eSpCas9(1.1)	1	25113	1206	4.80%
			2	15316	1036	6.76%

		SpCas9-HF1	1	37057	720	1.94%
			2	20118	1984	9.86%
	Control		1	34310	27	0.08%
			2	36667	27	0.07%
Site 4	U3-sgRNA-AN$_{20}$	WT SpCas9	1	22043	2836	12.87%
			2	19523	1240	6.35%
		eSpCas9(1.0)	1	14792	138	0.93%
			2	9312	35	0.38%
		eSpCas9(1.1)	1	14084	264	1.87%
			2	23291	139	0.60%
		SpCas9-HF1	1	43261	166	0.38%
			2	25883	50	0.19%
	U3:tRNA-sgRNA-N$_{20}$	WT SpCas9	1	17438	2751	15.78%
			2	21302	2004	9.41%
		eSpCas9(1.0)	1	35365	4772	13.49%
			2	32606	1288	3.95%
		eSpCas9(1.1)	1	8509	1708	20.07%
			2	18087	1477	8.17%
		SpCas9-HF1	1	15306	2420	15.81%
			2	23584	1749	7.42%
	U6:sgRNA-GN$_{20}$	WT SpCas9	1	60349	5221	8.65%
			2	53263	3365	6.32%
		eSpCas9(1.0)	1	160493	2351	1.46%
			2	174897	3289	1.88%
		eSpCas9(1.1)	1	65752	2224	3.38%
			2	50299	1707	3.39%
		SpCas9-HF1	1	131037	3169	2.42%
			2	123036	3790	3.08%
	U6:sgRNA-GN$_{19}$	WT SpCas9	1	36922	2716	7.36%
			2	21947	2004	9.13%
		eSpCas9(1.0)	1	19076	876	4.59%
			2	13845	703	5.08%
		eSpCas9(1.1)	1	126777	9875	7.79%
			2	104752	9108	8.69%
		SpCas9-HF1	1	148352	7666	5.17%
			2	120336	7162	5.95%
	Control		1	23382	59	0.25%
			2	14806	29	0.20%
Site 5	U3-sgRNA-AN$_{20}$	WT SpCas9	1	4931	397	8.05%
			2	16135	1331	8.25%
		eSpCas9(1.0)	1	4060	67	1.65%
			2	12066	178	1.48%
		eSpCas9(1.1)	1	9224	74	0.80%
			2	28489	302	1.06%
		SpCas9-HF1	1	13775	69	0.50%
			2	34016	255	0.75%
	U3:tRNA-sgRNA-N$_{20}$	WT SpCas9	1	6935	569	8.20%
			2	15805	1758	11.12%
		eSpCas9(1.0)	1	22149	2053	9.27%
			2	30298	2317	7.65%
		eSpCas9(1.1)	1	6627	707	10.67%
			2	14403	1378	9.57%
		SpCas9-HF1	1	12027	1741	14.48%
			2	21781	2677	12.29%
	Control		1	46453	11	0.02%
			2	30642	7	0.02%
Site 6	U3-sgRNA-AN$_{19}$	WT SpCas9	1	1165	104	8.93%
			2	1858	230	12.38%

		eSpCas9(1.0)	1	15826	647	4.09%
			2	24802	1116	4.50%
		eSpCas9(1.1)	1	6176	252	4.08%
			2	5705	353	6.19%
		SpCas9-HF1	1	14039	560	3.99%
			2	13791	670	4.86%
	U3-sgRNA-AN$_{20}$	WT SpCas9	1	857	55	6.42%
			2	5815	343	5.90%
		eSpCas9(1.0)	1	17426	209	1.20%
			2	47642	699	1.47%
		eSpCas9(1.1)	1	6143	33	0.54%
			2	12253	163	1.33%
		SpCas9-HF1	1	8172	7	0.09%
			2	63632	56	0.09%
	U3:tRNA-sgRNA-N$_{20}$	WT SpCas9	1	6595	834	12.65%
			2	14051	1831	13.03%
		eSpCas9(1.0)	1	42245	2332	5.52%
			2	63620	2764	4.34%
		eSpCas9(1.1)	1	10836	566	5.22%
			2	21807	1373	6.30%
		SpCas9-HF1	1	70313	4500	6.40%
			2	83009	2360	2.84%
	Control		1	77479	39	0.05%
			2	64881	27	0.04%
Site 7	U3-sgRNA-AN$_{19}$	WT SpCas9	1	15665	3070	19.60%
			2	35866	5984	16.68%
		eSpCas9(1.0)	1	10392	1058	10.18%
			2	24177	2727	11.28%
		eSpCas9(1.1)	1	21251	4095	19.27%
			2	35509	7229	20.36%
		SpCas9-HF1	1	12840	1850	14.41%
			2	33796	2516	7.44%
	U3-sgRNA-AN$_{20}$	WT SpCas9	1	70994	2882	4.06%
			2	85169	5104	5.99%
		eSpCas9(1.0)	1	23706	741	3.13%
			2	38972	1217	3.12%
		eSpCas9(1.1)	1	10343	162	1.57%
			2	16566	862	5.20%
		SpCas9-HF1	1	240298	5480	2.28%
			2	303900	11160	3.67%
	U3:tRNA-sgRNA-N$_{20}$	WT SpCas9	1	11379	1810	15.91%
			2	28061	5980	21.31%
		eSpCas9(1.0)	1	13234	876	6.62%
			2	31823	2773	8.71%
		eSpCas9(1.1)	1	10370	2060	19.87%
			2	19382	5040	26.00%
		SpCas9-HF1	1	8566	1013	11.83%
			2	14807	3233	21.83%
	Control		1	104244	91	0.09%
			2	96506	57	0.06%
OT2-1	U3:tRNA-sgRNA-AN$_{20}$	WT SpCas9	1	19441	696	3.58%
			2	33621	2528	7.52%
		eSpCas9(1.0)	1	8747	58	0.66%
			2	31177	342	1.10%
		eSpCas9(1.1)	1	9393	155	1.65%
			2	62235	2231	3.58%
		SpCas9-HF1	1	58519	451	0.77%
			2	138021	1232	0.89%

	Control		1	399	1	0.25%
			2	1350	6	0.44%
OT2-2	U3:tRNA-sgRNA-AN$_{20}$	WT SpCas9	1	11375	393	3.45%
			2	7298	385	5.28%
		eSpCas9(1.0)	1	2562	18	0.70%
			2	43257	161	0.37%
		eSpCas9(1.1)	1	8142	55	0.68%
			2	23440	86	0.37%
		SpCas9-HF1	1	3246	11	0.34%
			2	32830	16	0.05%
	Control		1	1283	5	0.39%
			2	323	2	0.62%
OT6-1	U3-sgRNA-AN$_{19}$	WT SpCas9	1	4447	261	5.87%
			2	5026	189	3.76%
		eSpCas9(1.0)	1	16198	469	2.90%
			2	21985	1036	4.71%
		eSpCas9(1.1)	1	9488	290	3.06%
			2	10374	541	5.22%
		SpCas9-HF1	1	32522	53	0.16%
			2	62635	1545	2.47%
	U3:tRNA-sgRNA-N$_{20}$	WT SpCas9	1	12901	1534	11.89%
			2	13718	1055	7.69%
		eSpCas9(1.0)	1	110496	6337	5.74%
			2	121321	6045	4.98%
		eSpCas9(1.1)	1	22816	824	3.61%
			2	25131	1332	5.30%
		SpCas9-HF1	1	64456	73	0.11%
			2	77605	2015	2.60%
	Control		1	110844	147	0.13%
OT6-2	U3-sgRNA-AN$_{19}$	WT SpCas9	1	3273	105	3.21%
			2	2134	93	4.36%
		eSpCas9(1.0)	1	16016	1	0.01%
			2	11981	2	0.02%
		eSpCas9(1.1)	1	9782	1	0.01%
			2	6843	6	0.09%
		SpCas9-HF1	1	27923	3	0.01%
			2	43073	4	0.01%
	U3:tRNA-sgRNA-N$_{20}$	WT SpCas9	1	6779	270	3.98%
			2	6729	441	6.55%
		eSpCas9(1.0)	1	46700	35	0.07%
			2	35818	26	0.07%
		eSpCas9(1.1)	1	14362	18	0.13%
			2	11005	8	0.07%
		SpCas9-HF1	1	33681	9	0.03%
			2	21682	2	0.01%
	Control		1	66279	1	0.00%
			2	49448	18	0.04%
OT6-3	U3-sgRNA-AN$_{19}$	WT SpCas9	1	4219	212	5.02%
			2	2037	203	9.97%
		eSpCas9(1.0)	1	12839	23	0.18%
			2	6893	21	0.30%
		eSpCas9(1.1)	1	3452	9	0.26%
			2	1757	7	0.40%
		SpCas9-HF1	1	22702	60	0.26%
			2	16277	27	0.17%
	U3:tRNA-sgRNA-N$_{20}$	WT SpCas9	1	12527	817	6.52%
			2	8336	804	9.64%
		eSpCas9(1.0)	1	67711	124	0.18%

			2	60479	151	0.25%
		eSpCas9(1.1)	1	29812	53	0.18%
			2	13964	29	0.21%
		SpCas9-HF1	1	42227	86	0.20%
			2	31243	54	0.17%
	Control		1	38022	66	0.17%
			2	36681	71	0.19%
Site 2 on-taget	WT SpCas9			29678	7113	23.97%
	eSpCas9(1.0)			87858	12888	14.67%
	eSpCas9(1.1)			23134	2581	11.16%
	SpCas9-HF1			15886	2878	18.12%
Site 2-mm-19-20	WT SpCas9			6385	516	8.08%
	eSpCas9(1.0)			38296	489	1.28%
	eSpCas9(1.1)			1700	22	1.29%
	SpCas9-HF1			73206	197	0.27%
Site 2-mm-18-19	WT SpCas9			64112	5306	8.28%
	eSpCas9(1.0)			25346	79	0.31%
	eSpCas9(1.1)			24268	35	0.14%
	SpCas9-HF1			23040	16	0.07%
Site 2-mm-17-18	WT SpCas9			14586	615	4.22%
	eSpCas9(1.0)			63322	49	0.08%
	eSpCas9(1.1)			48426	349	0.72%
	SpCas9-HF1			35956	32	0.09%
Site 2-mm-16-17	WT SpCas9			12883	463	3.59%
	eSpCas9(1.0)			24965	23	0.09%
	eSpCas9(1.1)			46893	47	0.10%
	SpCas9-HF1			2326	1	0.04%
Site 2-mm-15-16	WT SpCas9			12804	737	5.76%
	eSpCas9(1.0)			53436	57	0.11%
	eSpCas9(1.1)			10652	9	0.08%
	SpCas9-HF1			6410	7	0.11%
Site 2-mm-14-15	WT SpCas9			13413	255	1.90%
	eSpCas9(1.0)			41069	88	0.21%
	eSpCas9(1.1)			3824	12	0.31%
	SpCas9-HF1			66842	611	0.91%
Site 2-mm-13-14	WT SpCas9			59351	315	0.53%
	eSpCas9(1.0)			30101	85	0.28%
	eSpCas9(1.1)			30688	15	0.05%
	SpCas9-HF1			30252	33	0.11%
Site 2-mm-12-13	WT SpCas9			28377	512	1.80%
	eSpCas9(1.0)			74871	266	0.36%
	eSpCas9(1.1)			77547	874	1.13%
	SpCas9-HF1			53502	56	0.10%
Site 2-mm-11-12	WT SpCas9			21389	674	3.15%
	eSpCas9(1.0)			36632	417	1.14%
	eSpCas9(1.1)			45312	380	0.84%
	SpCas9-HF1			4349	26	0.60%
Site 2-mm-10-11	WT SpCas9			20542	3798	18.49%
	eSpCas9(1.0)			53635	5769	10.76%
	eSpCas9(1.1)			12371	1111	8.98%
	SpCas9-HF1			9951	765	7.69%
Site 2-mm-9-10	WT SpCas9			14562	410	2.82%
	eSpCas9(1.0)			16639	471	2.83%
	eSpCas9(1.1)			4136	79	1.91%
	SpCas9-HF1			31888	203	0.64%
	WT SpCas9			88657	111	0.13%
	eSpCas9(1.0)			50541	65	0.13%

Site 2-mm-8-9	eSpCas9(1.1)		20932	17	0.08%
	SpCas9-HF1		24658	18	0.07%
Site 2-mm-7-8	WT SpCas9		15373	102	0.66%
	eSpCas9(1.0)		42727	80	0.19%
	eSpCas9(1.1)		43930	218	0.50%
	SpCas9-HF1		21688	123	0.57%
Site 2-mm-6-7	WT SpCas9		15954	698	4.38%
	eSpCas9(1.0)		20748	218	1.05%
	eSpCas9(1.1)		33745	548	1.62%
	SpCas9-HF1		7724	21	0.27%
Site 2-mm-5-6	WT SpCas9		16336	28	0.17%
	eSpCas9(1.0)		51441	64	0.12%
	eSpCas9(1.1)		13404	20	0.15%
	SpCas9-HF1		9993	13	0.13%
Site 2-mm-4-5	WT SpCas9		14863	114	0.77%
	eSpCas9(1.0)		65291	38	0.06%
	eSpCas9(1.1)		4890	2	0.04%
	SpCas9-HF1		84045	64	0.07%
Site 2-mm-3-4	WT SpCas9		78006	200	0.26%
	eSpCas9(1.0)		44634	168	0.38%
	eSpCas9(1.1)		52593	68	0.13%
	SpCas9-HF1		45272	44	0.10%
Site 2-mm-2-3	WT SpCas9		35707	108	0.30%
	eSpCas9(1.0)		79249	152	0.19%
	eSpCas9(1.1)		81830	1006	1.23%
	SpCas9-HF1		78929	86	0.11%
Site 2-mm-1-2	WT SpCas9		30374	65	0.21%
	eSpCas9(1.0)		36220	45	0.12%
	eSpCas9(1.1)		69625	108	0.16%
	SpCas9-HF1		7870	4	0.05%
control			18917	13	0.07%

Table S3. Guide sequences containing pairs of mismatches at successive positions of Site 2, and oligos used to construct U3:tRNA-sgRNA expression vectors (the U3:tRNA-sgRNA fragment containing two BsaI restriction sites was synthesized and cloned into pUC57). More information of OsU3:tRNA-sgRNA-N_{20} expression constructs can be found in Supplementary Fig. 1b.

Name	Guide sequences containing pairs of mismatches	Oligo (5'-3')
Site 2	GACGTCGGCGAGGAAGGCCT	F:TGCAGACGTCGGCGAGGAAGGCCT<br>R:AAACAGGCCTTCCTCGCCGACGTC
Mismatch-1	AGCGTCGGCGAGGAAGGCCT	F:TGCAAGCGTCGGCGAGGAAGGCCT<br>R:AAACAGGCCTTCCTCGCCGACGCT
Mismatch-2	GGTGTCGGCGAGGAAGGCCT	F:TGCAGGTGTCGGCGAGGAAGGCCT<br>R:AAACAGGCCTTCCTCGCCGACACC
Mismatch-3	GATATCGGCGAGGAAGGCCT	F:TGCAGATATCGGCGAGGAAGGCCT<br>R:AAACAGGCCTTCCTCGCCGATATC
Mismatch-4	GACACCGGCGAGGAAGGCCT	F:TGCAGACACCGGCGAGGAAGGCCT<br>R:AAACAGGCCTTCCTCGCCGGTGTC
Mismatch-5	GACGCTGGCGAGGAAGGCCT	F:TGCAGACGCTGGCGAGGAAGGCCT<br>R:AAACAGGCCTTCCTCGCCAGCGTC
Mismatch-6	GACGTTAGCGAGGAAGGCCT	F:TGCAGACGTTAGCGAGGAAGGCCT<br>R:AAACAGGCCTTCCTCGCTAACGTC
Mismatch-7	GACGTCAACGAGGAAGGCCT	F:TGCAGACGTCAACGAGGAAGGCCT<br>R:AAACAGGCCTTCCTCGTTGACGTC
Mismatch-8	GACGTCGATGAGGAAGGCCT	F:TGCAGACGTCGATGAGGAAGGCCT<br>R:AAACAGGCCTTCCTCATCGACGTC
Mismatch-9	GACGTCGGTAAGGAAGGCCT	F:TGCAGACGTCGGTAAGGAAGGCCT<br>R:AAACAGGCCTTCCTTACCGACGTC
Mismatch-10	GACGTCGGCAGGGAAGGCCT	F:TGCAGACGTCGGCAGGGAAGGCCT<br>R:AAACAGGCCTTCCCTGCCGACGTC
Mismatch-11	GACGTCGGCGGAGAAGGCCT	F:TGCAGACGTCGGCGGAGAAGGCCT<br>R:AAACAGGCCTTCTCCGCCGACGTC
Mismatch-12	GACGTCGGCGAAAAAGGCCT	F:TGCAGACGTCGGCGAAAAAGGCCT<br>R:AAACAGGCCTTTTTCGCCGACGTC
Mismatch-13	GACGTCGGCGAGAGAGGCCT	F:TGCAGACGTCGGCGAGAGAGGCCT<br>R:AAACAGGCCTCTCTCGCCGACGTC
Mismatch-14	GACGTCGGCGAGGGGGGCCT	F:TGCAGACGTCGGCGAGGGGGGCCT<br>R:AAACAGGCCCCCCTCGCCGACGTC
Mismatch-15	GACGTCGGCGAGGAGAGCCT	F:TGCAGACGTCGGCGAGGAGAGCCT<br>R:AAACAGGCTCTCCTCGCCGACGTC
Mismatch-16	GACGTCGGCGAGGAAAACCT	F:TGCAGACGTCGGCGAGGAAAACCT<br>R:AAACAGGTTTTCCTCGCCGACGTC
Mismatch-17	GACGTCGGCGAGGAAGATCT	F:TGCAGACGTCGGCGAGGAAGATCT<br>R:AAACAGATCTTCCTCGCCGACGTC
Mismatch-18	GACGTCGGCGAGGAAGGTTT	F:TGCAGACGTCGGCGAGGAAGGTTT<br>R:AAACAAACCTTCCTCGCCGACGTC
Mismatch-19	GACGTCGGCGAGGAAGGCTC	F:TGCAGACGTCGGCGAGGAAGGCTC<br>R:AAACGAGCCTTCCTCGCCGACGTC

The mismatches sites are highlighted in red.

Table S4. PCR primers used in this study.

Primer name	Sequence (5'-3')	Application
Site 1-F	AGAAAGGGGAGAAACGCTTC	amplifying target sites
Site 1-R	TCACCTACCACAACACCAAC	
Site 2-F	GGCTCATGTAGGCGATGGTC	
Site 2-R	CGCGCTCAAGGTGCTCTA	
Site 3-F	TGCCGCACCATCCACACCGTCC	
Site 3-R	CTTCTTCCTCTCCCTGCCCCCG	
Site 4-F	TAGGCAACATGTCACTTGGCTCTAGAG	
Site 4-R	CTCCACTACAGACTGAGCACAAAGCTTC	
Site 5-F	CAAGACAGAAGAAATGGCACT	
Site 5-R	AGAAATCGTTGGTAAAGGAAG	
Site 6-F	CAAAAAGCGCCCGTTAGAGCG	
Site 6-R	GCGGATGAACTTCTTGAGGTC	
Site 7-F	AGGCTGATTCGTTGTGTTCCTG	
Site 7-R	CTCCCCACTTCCTTATCTCGTT	
OT2-1-F	GGGAGCGGGTTCACCGGCATCTC	
OT2-1-R	TGTCGTGTCAGGTCGGTAAGACC	
OT2-2-F	ACCAAACCAAAACCCACACACCC	
OT2-2-R	TCAGCATCCCCGCCCAACATCTC	
OT6-1-F	ATGCCACAAGCCCAACCCAATTCATCCCCA	
OT6-1-R	TGCAGCCTGCACGGCACAATCCAAATTCCCA	
OT6-2-F	ACGCTCCTCCCCCATTTCAAATC	
OT6-2-R	AGAAGCGGAGAAGACACGGGATAATCAGGCA	
OT6-3-F	CCTCCCACTTCTCCGCCTCC	
OT6-3-R	TCAATGTATTGCCCACATCA	
qPCR-UBI-F	TGGTCAGTAATCAGCCAGTTTG	Quantitative RT-PCR
qPCR-UBI-R	CAAATACTTGACGAACAGAGGC	
qPCR-sgRNA-F	GTTTTAGAGCTAGAAATAGCAAGT	
qPCR-sgRNA-R	TCGGTGCCACTTTTTCAAGTTGA	
sgRNA-RT	ACTCGGTGCCACTTTTTCAA	Reverse transcription for sgRNA
L5AD5-F	CGGGTCTCAGGCAGGATGGGCAGTCTGGGCAACAAAGCACCAGTGG	construct pre-tRNA-sgRNA expression cassettes
L3AD5-R	TAGGTCTCCAAACGGATGAGCGACAGCAAACAAAAAAAAAAGCACCGACTCG	
S5AD5-F	CGGGTCTCAGGCAGGATGGGCAGTCTGGGCA	
S3AD5-R	TAGGTCTCCAAACGGATGAGCGACAGCAAAC	
K810A-F	CACGCAGTTGCAAAATGAGGCCCTCTATCTGTACTATC	Site-directed mutagenesis of WT SpCas9
K810A-R	GGCCTCATTTTGCAACTGCGTGTTTTCCACCGGGTGCT	
K1003A-F	ATTGATTAAGAAGTACCCGGCGCTCGAGTCCGAATTCG	
K1003A-R	CGCCGGGTACTTCTTAATCAATGCTGTTCCGACCACAG	
R1060A-F	TAATGGCGAAATCCGGAAGGCGCCACTTATTGAGACCA	
R1060A-R	CGCCTTCCGGATTTCGCCATTAGCCAGCGTGATCTCAG	
K848A-F	CGTTCCTCAGTCCTTCCTTGCGGATGACAGCATTGACA	

K848A-R	CGCAAGGAAGGACTGAGGAACGATATGATCGACGTCGT	
N497A-F	CTTCATTGAGCGCATGACAGCCTTTGACAAGAACCTGCC	
N497A-R	GGCAGGTTCTTGTCAAAGGCTGTCATGCGCTCAATGAAG	
R661A-F	AAGATACACCGGATGGGGAGCCCTGTCCCGCAAGCTTAT	
R661A-R	ATAAGCTTGCGGGACAGGGCTCCCCATCCGGTGTATCTT	
Q695A-F	CGCGAACAGGAATTTTATGGCCCTGATTCACGATGACTC	
Q695A-R	GAGTCATCGTGAATCAGGGCCATAAAATTCCTGTTCGCG	
Q926A-F	GCAACTGGTTGAGACTAGAGCCATCACGAAGCACGTGGC	
Q926A-R	GCCACGTGCTTCGTGATGGCTCTAGTCTCAACCAGTTGC	

Table S5. Second round PCR primers with barcodes for deep amplicon sequencing.

	F (5'-3')	R (5'-3')
Site 1	CAGATCAGCAACCACCACCAGCAC	GCCTAAGGGATCTTACCCCGAGGAT
	GGCTACAGCAACCACCACCAGCAC	TCAAGTGGGATCTTACCCCGAGGAT
	AGTTCCAGCAACCACCACCAGCAC	CTGATCGGGATCTTACCCCGAGGAT
	GTAGAGAGCAACCACCACCAGCAC	AAGCTAGGGATCTTACCCCGAGGAT
	CGATGTAGCAACCACCACCAGCAC	CACTGTGGGATCTTACCCCGAGGAT
	ACTGATAGCAACCACCACCAGCAC	AGGAATGGGATCTTACCCCGAGGAT
	CCAACAAGCAACCACCACCAGCAC	ATTCCGGGGATCTTACCCCGAGGAT
	CTATACAGCAACCACCACCAGCAC	GAATGAGGGATCTTACCCCGAGGAT
	TGACCAAGCAACCACCACCAGCAC	CGTGATGGGATCTTACCCCGAGGAT
Site 2	CGATGTTTGGATGGCTTGATGTCG	TGCCGAGATCCTGCTCGAGTACAT
	ATCACGTTGGATGGCTTGATGTCG	CTTCGAGATCCTGCTCGAGTACAT
	TTAGGCTTGGATGGCTTGATGTCG	TCGGGAGATCCTGCTCGAGTACAT
	ACTTGATTGGATGGCTTGATGTCG	GAATGAGATCCTGCTCGAGTACAT
	GTCCGCTTGGATGGCTTGATGTCG	TGTTGGGATCCTGCTCGAGTACAT
	GTGGCCTTGGATGGCTTGATGTCG	AAAATGGATCCTGCTCGAGTACAT
	GTTTCGTTGGATGGCTTGATGTCG	GCCATGGATCCTGCTCGAGTACAT
	CGTACGTTGGATGGCTTGATGTCG	CGCCTGGATCCTGCTCGAGTACAT
	ATTCCTTTGGATGGCTTGATGTCG	CTTTTGGATCCTGCTCGAGTACAT
Site 3	CAGATCGCGGGACCTCACGTCGCT	GCCTAAGCTCCAGCTCCGACAGCG
	GGCTACGCGGGACCTCACGTCGCT	TCAAGTGCTCCAGCTCCGACAGCG
	AGTTCCGCGGGACCTCACGTCGCT	CTGATCGCTCCAGCTCCGACAGCG
	GTAGAGGCGGGACCTCACGTCGCT	AAGCTAGCTCCAGCTCCGACAGCG
	CGATGTGCGGGACCTCACGTCGCT	CACTGTGCTCCAGCTCCGACAGCG
	ACTGATGCGGGACCTCACGTCGCT	AGGAATGCTCCAGCTCCGACAGCG
	CCAACAGCGGGACCTCACGTCGCT	ATTCCGGCTCCAGCTCCGACAGCG
	CTATACGCGGGACCTCACGTCGCT	GAATGAGCTCCAGCTCCGACAGCG
	TGACCAGCGGGACCTCACGTCGCT	CGTGATGCTCCAGCTCCGACAGCG
Site 4	CAGATCCGGTTTTCTTTCCTTTTG	GCCTAATCTTGCATAGTTGCTTCG
	GGCTACCGGTTTTCTTTCCTTTTG	TCAAGTTCTTGCATAGTTGCTTCG
	AGTTCCCGGTTTTCTTTCCTTTTG	CTGATCTCTTGCATAGTTGCTTCG
	GTAGAGCGGTTTTCTTTCCTTTTG	AAGCTATCTTGCATAGTTGCTTCG
	CGATGTCGGTTTTCTTTCCTTTTG	CACTGTTCTTGCATAGTTGCTTCG
	ACTGATCGGTTTTCTTTCCTTTTG	AGGAATTCTTGCATAGTTGCTTCG
	CCAACACGGTTTTCTTTCCTTTTG	ATTCCGTCTTGCATAGTTGCTTCG
	CTATACCGGTTTTCTTTCCTTTTG	GAATGATCTTGCATAGTTGCTTCG
	TGACCACGGTTTTCTTTCCTTTTG	CGTGATTCTTGCATAGTTGCTTCG
Site 5	CAGATCTGCTAGGATCCTCCGTGAGA	GCCTAAAGCCTTAATAACCTGGTGG
	GGCTACTGCTAGGATCCTCCGTGAGA	TCAAGTAGCCTTAATAACCTGGTGG
	AGTTCCTGCTAGGATCCTCCGTGAGA	CTGATCAGCCTTAATAACCTGGTGG
	GTAGAGTGCTAGGATCCTCCGTGAGA	AAGCTAAGCCTTAATAACCTGGTGG
	CGATGTTGCTAGGATCCTCCGTGAGA	CACTGTAGCCTTAATAACCTGGTGG
	ACTGATTGCTAGGATCCTCCGTGAGA	AGGAATAGCCTTAATAACCTGGTGG
	CCAACATGCTAGGATCCTCCGTGAGA	ATTCCGAGCCTTAATAACCTGGTGG
	CTATACTGCTAGGATCCTCCGTGAGA	GAATGAAGCCTTAATAACCTGGTGG
	TGACCATGCTAGGATCCTCCGTGAGA	CGTGATAGCCTTAATAACCTGGTGG
Site 6	TGACCAGGTGACGACGACGACGGAC	CGTGATCTTCTTCAGCGCCACGATC
	CAGATCGGTGACGACGACGACGGAC	GCCTAACTTCTTCAGCGCCACGATC
	GGCTACGGTGACGACGACGACGGAC	TCAAGTCTTCTTCAGCGCCACGATC
	AGTTCCGGTGACGACGACGACGGAC	CTGATCCTTCTTCAGCGCCACGATC
	GTAGAGGGTGACGACGACGACGGAC	AAGCTACTTCTTCAGCGCCACGATC
	CGATGTGGTGACGACGACGACGGAC	CACTGTCTTCTTCAGCGCCACGATC

	ACTGATGGTGACGACGACGACGGAC	AGGAATCTTCTTCAGCGCCACGATC
	CCAACAGGTGACGACGACGACGGAC	ATTCCGCTTCTTCAGCGCCACGATC
	CTATACGGTGACGACGACGACGGAC	GAATGACTTCTTCAGCGCCACGATC
Site 7	CAGATCCAAGAAGATTAATGACAT	GCCTAAAAACAACATAAATGTCCT
	GGCTACCAAGAAGATTAATGACAT	TCAAGTAAACAACATAAATGTCCT
	AGTTCCCAAGAAGATTAATGACAT	CTGATCAAACAACATAAATGTCCT
	GTAGAGCAAGAAGATTAATGACAT	AAGCTAAAACAACATAAATGTCCT
	CGATGTCAAGAAGATTAATGACAT	CACTGTAAACAACATAAATGTCCT
	ACTGATCAAGAAGATTAATGACAT	AGGAATAAACAACATAAATGTCCT
	CCAACACAAGAAGATTAATGACAT	ATTCCGAAACAACATAAATGTCCT
	CTATACCAAGAAGATTAATGACAT	GAATGAAACAACATAAATGTCCT
	TGACCACAAGAAGATTAATGACAT	CGTGATAAACAACATAAATGTCCT
	AGTCAACAAGAAGATTAATGACAT	TAGCGCAAACAACATAAATGTCCT
	CCGTCCCAAGAAGATTAATGACAT	AGCTAGAAACAACATAAATGTCCT
	CGTACGCAAGAAGATTAATGACAT	CGCCTGAAACAACATAAATGTCCT
	GGTAGCCAAGAAGATTAATGACAT	ATCGTGAAACAACATAAATGTCCT
OT2-1	CGATGTAGGGAGACGTGGGTCCGC	CACTGTGCGTGGTTCTCGGCTTCG
	ACTGATAGGGAGACGTGGGTCCGC	AGGAATGCGTGGTTCTCGGCTTCG
	CCAACAAGGGAGACGTGGGTCCGC	ATTCCGGCGTGGTTCTCGGCTTCG
	CTATACAGGGAGACGTGGGTCCGC	GAATGAGCGTGGTTCTCGGCTTCG
	TCGAAGAGGGAGACGTGGGTCCGC	TGCCGAGCGTGGTTCTCGGCTTCG
	CAGATCAGGGAGACGTGGGTCCGC	GCCTAAGCGTGGTTCTCGGCTTCG
	GGCTACAGGGAGACGTGGGTCCGC	TCAAGTGCGTGGTTCTCGGCTTCG
	AGTTCCAGGGAGACGTGGGTCCGC	CTGATCGCGTGGTTCTCGGCTTCG
	GTAGAGAGGGAGACGTGGGTCCGC	AAGCTAGCGTGGTTCTCGGCTTCG
OT2-2	CGATGTATGGCGTCGCTGTTCGG	CACTGTAGACGTACGACGCCGA
	ACTGATATGGCGTCGCTGTTCGG	AGGAATAGACGTACGACGCCGA
	CCAACAATGGCGTCGCTGTTCGG	ATTCCGAGACGTACGACGCCGA
	CTATACATGGCGTCGCTGTTCGG	GAATGAAGACGTACGACGCCGA
	TCGAAGATGGCGTCGCTGTTCGG	TGCCGAAGACGTACGACGCCGA
OT6-1	TGACCATGCTGATGATGAGTGTG	CGTGATGCTCCAGGCGGATCTTCTTG
	CAGATCTGCTGATGATGAGTGTG	GCCTAAGCTCCAGGCGGATCTTCTTG
	GGCTACTGCTGATGATGAGTGTG	TCAAGTGCTCCAGGCGGATCTTCTTG
	AGTTCCTGCTGATGATGAGTGTG	CTGATCGCTCCAGGCGGATCTTCTTG
	GTAGAGTGCTGATGATGAGTGTG	AAGCTAGCTCCAGGCGGATCTTCTTG
	CGATGTTGCTGATGATGAGTGTG	CACTGTGCTCCAGGCGGATCTTCTTG
	ACTGATTGCTGATGATGAGTGTG	AGGAATGCTCCAGGCGGATCTTCTTG
	CCAACATGCTGATGATGAGTGTG	ATTCCGGCTCCAGGCGGATCTTCTTG
	CTATACTGCTGATGATGAGTGTG	GAATGAGCTCCAGGCGGATCTTCTTG
OT6-2	TGACCACCAGCCTCGTCTTCTTCA	CGTGATGCCGAATCTAGGGTTCAT
	CAGATCCCAGCCTCGTCTTCTTCA	GCCTAAGCCGAATCTAGGGTTCAT
	GGCTACCCAGCCTCGTCTTCTTCA	TCAAGTGCCGAATCTAGGGTTCAT
	AGTTCCCCAGCCTCGTCTTCTTCA	CTGATCGCCGAATCTAGGGTTCAT
	GTAGAGCCAGCCTCGTCTTCTTCA	AAGCTAGCCGAATCTAGGGTTCAT
	CGATGTCCAGCCTCGTCTTCTTCA	CACTGTGCCGAATCTAGGGTTCAT
	ACTGATCCAGCCTCGTCTTCTTCA	AGGAATGCCGAATCTAGGGTTCAT
	CCAACACCAGCCTCGTCTTCTTCA	ATTCCGGCCGAATCTAGGGTTCAT

	CTATACCCAGCCTCGTCTTCTTCA	GAATGAGCCGAATCTAGGGTTCAT
OT6-3	TGACCACGGGGCGCAGTACGAGAA	CGTGATCGCCCTCATCCTCCTGCTC
	CAGATCCGGGGCGCAGTACGAGAA	GCCTAACGCCCTCATCCTCCTGCTC
	GGCTACCGGGGCGCAGTACGAGAA	TCAAGTCGCCCTCATCCTCCTGCTC
	AGTTCCCGGGGCGCAGTACGAGAA	CTGATCCGCCCTCATCCTCCTGCTC
	GTAGAGCGGGGCGCAGTACGAGAA	AAGCTACGCCCTCATCCTCCTGCTC
	CGATGTCGGGGCGCAGTACGAGAA	CACTGTCGCCCTCATCCTCCTGCTC
	ACTGATCGGGGCGCAGTACGAGAA	AGGAATCGCCCTCATCCTCCTGCTC
	CCAACACGGGGCGCAGTACGAGAA	ATTCCGCGCCCTCATCCTCCTGCTC
	CTATACCGGGGCGCAGTACGAGAA	GAATGACGCCCTCATCCTCCTGCTC
Mismatch at Site 2	CGATGTTTGGATGGCTTGATGTCG	TGCCGAGATCCTGCTCGAGTACAT
	ATCACGTTGGATGGCTTGATGTCG	CTTCGAGATCCTGCTCGAGTACAT
	TTAGGCTTGGATGGCTTGATGTCG	TCGGGAGATCCTGCTCGAGTACAT
	ACTTGATTGGATGGCTTGATGTCG	GAATGAGATCCTGCTCGAGTACAT
	GATCAGTTGGATGGCTTGATGTCG	ATTATAGATCCTGCTCGAGTACAT
	TAGCTTTTGGATGGCTTGATGTCG	GCTGTAGATCCTGCTCGAGTACAT
	GGCTACTTGGATGGCTTGATGTCG	CGATTAGATCCTGCTCGAGTACAT
	AGTCAATTGGATGGCTTGATGTCG	TAGCGCGATCCTGCTCGAGTACAT
	AGTTCCTTGGATGGCTTGATGTCG	TCTGAGGATCCTGCTCGAGTACAT
	ATGTCATTGGATGGCTTGATGTCG	GTATAGGATCCTGCTCGAGTACAT
	CCGTCCTTGGATGGCTTGATGTCG	AGCTAGGATCCTGCTCGAGTACAT
	GTAGAGTTGGATGGCTTGATGTCG	ATTCCGGATCCTGCTCGAGTACAT
	GTCCGCTTGGATGGCTTGATGTCG	TGTTGGGATCCTGCTCGAGTACAT
	GTGGCCTTGGATGGCTTGATGTCG	AAAATGGATCCTGCTCGAGTACAT
	GTTTCGTTGGATGGCTTGATGTCG	GCCATGGATCCTGCTCGAGTACAT
	CGTACGTTGGATGGCTTGATGTCG	CGCCTGGATCCTGCTCGAGTACAT
	GAGTGGTTGGATGGCTTGATGTCG	TGAGTGGATCCTGCTCGAGTACAT
	GGTAGCTTGGATGGCTTGATGTCG	ATCGTGGATCCTGCTCGAGTACAT
	ACTGATTTGGATGGCTTGATGTCG	CCGGTGGATCCTGCTCGAGTACAT
	ATGAGCTTGGATGGCTTGATGTCG	TAGTTGGATCCTGCTCGAGTACAT
	ATTCCTTTGGATGGCTTGATGTCG	CTTTTGGATCCTGCTCGAGTACAT
