# Supplementary material for: Perfectly matched 20-nucleotide guide RNA sequences enable robust genome editing using high-fidelity SpCas9 nucleases
Source: Genome Biol. 2017 Oct 11;18:191. doi: 10.1186/s13059-017-1325-9 (PMC5637269; doi:10.1186/s13059-017-1325-9)
Supplement: Additional file 2: — Rice codon optimized DNA sequences of WT Cas9, eSpCas9(1.0), eSpCas9(1.1), and SpCas9-HF1. (PDF 296 kb) [file 13059_2017_1325_MOESM2_ESM.pdf]

Sequences. Rice codon optimized DNA sequences of WT Cas9, eSpCas9(1.0) (K810A, K1003A, R1060A), eSpCas9(1.1) (K848A, K1003A, R1060A) and SpCas9-HF1(N497A, R661A, Q695A, Q926A). The rice codon optimized SpCas9 and three variants are colored in blue and the modified codons are colored in red. The NLSs are underlined.

WT SpCas9

ATGGCCCCTAAGAAGAAGAGAAAGGTCGGTATTCACGGCGTTCCTGCGGCGATGGACAAGAAGTA
TAGTATTGGTCTGGACATTGGGACGAATTCCGTTGGCTGGGCCGTGATCACCGATGAGTACAAGG
TCCCTTCCAAGAAGTTTAAGGTTCTGGGGAACACCGATCGGCACAGCATCAAGAAGAATCTCATT
GGAGCCCTCCTGTTCGACTCAGGCGAGACCGCCGAAGCAACAAGGCTCAAGAGAACCGCAAGGAG
ACGGTATACAAGAAGGAAGAATAGGATCTGCTACCTGCAGGAGATTTTCAGCAACGAAATGGCGA
AGGTGGACGATTCGTTCTTTCATAGATTGGAGGAGAGTTTCCTCGTCGAGGAAGATAAGAAGCAC
GAGAGGCATCCTATCTTTGGCAACATTGTCGACGAGGTTGCCTATCACGAAAAGTACCCCACAAT
CTATCATCTGCGGAAGAAGCTTGTGGACTCGACTGATAAGGCGGACCTTAGATTGATCTACCTCG
CTCTGGCACACATGATTAAGTTCAGGGGCCATTTTCTGATCGAGGGGGATCTTAACCCGGACAAT
AGCGATGTGGACAAGTTGTTCATCCAGCTCGTCCAAACCTACAATCAGCTCTTTGAGGAAAACCC
AATTAATGCTTCAGGCGTCGACGCCAAGGCGATCCTGTCTGCACGCCTTTCAAAGTCTCGCCGGC
TTGAGAACTTGATCGCTCAACTCCCGGGCGAAAAGAAGAACGGCTTGTTCGGGAATCTCATTGCA
CTTTCGTTGGGGCTCACACCAAACTTCAAGAGTAATTTTGATCTCGCTGAGGACGCAAAGCTGCA
GCTTTCCAAGGACACTTATGACGATGACCTGGATAACCTTTTGGCCCAAATCGGCGATCAGTACG
CGGACTTGTTCCTCGCCGCGAAGAATTTGTCGGACGCGATCCTCCTGAGTGATATTCTCCGCGTG
AACACCGAGATTACAAAGGCCCCGCTCTCGGCGAGTATGATCAAGCGCTATGACGAGCACCATCA
GGATCTGACCCTTTTGAAGGCTTTGGTCCGGCAGCAACTCCCAGAGAAGTACAAGGAAATCTTCT
TTGATCAATCCAAGAACGGCTACGCTGGTTATATTGACGGCGGGGCATCGCAGGAGGAATTCTAC
AAGTTTATCAAGCCAATTCTGGAGAAGATGGATGGCACAGAGGAACTCCTGGTGAAGCTCAATAG
GGAGGACCTTTTGCGGAAGCAAAGAACTTTCGATAACGGCAGCATCCCTCACCAGATTCATCTCG
GGGAGCTGCACGCCATCCTGAGAAGGCAGGAAGACTTCTACCCCTTTCTTAAGGATAACCGGGAG
AAGATCGAAAAGATTCTGACGTTCAGAATTCCGTACTATGTCGGACCACTCGCCCGGGGTAATTC
CAGATTTGCGTGGATGACCAGAAAGAGCGAGGAAACCATCACACCTTGGAACTTCGAGGAAGTGG
TCGATAAGGGCGCTTCCGCACAGAGCTTCATTGAGCGCATGACAAATTTTGACAAGAACCTGCCT
AATGAGAAGGTCCTTCCCAAGCATTCCCTCCTGTACGAGTATTTCACTGTTTATAACGAACTCAC
GAAGGTGAAGTATGTGACCGAGGGAATGCGCAAGCCCGCCTTCCTGAGCGGCGAGCAAAAGAAGG
CGATCGTGGACCTTTTGTTTAAGACCAATCGGAAGGTCACAGTTAAGCAGCTCAAGGAGGACTAC
TTCAAGAAGATTGAATGCTTCGATTCCGTTGAGATCAGCGGCGTGGAAGACAGGTTTAACGCGTC
ACTGGGGACTTACCACGATCTCCTGAAGATCATTAAGGATAAGGACTTCTTGGACAACGAGGAAA
ATGAGGATATCCTCGAAGACATTGTCCTGACTCTTACGTTGTTTGAGGATAGGGAAATGATCGAG
GAACGCTTGAAGACGTATGCCCATCTCTTCGATGACAAGGTTATGAAGCAGCTCAAGAGAAGAAG
ATACACCGGATGGGGAAGGCTGTCCCGCAAGCTTATCAATGGCATTAGAGACAAGCAATCAGGGA
AGACAATCCTTGACTTTTTGAAGTCTGATGGCTTCGCGAACAGGAATTTTATGCAGCTGATTCAC
GATGACTCACTTACTTTCAAGGAGGATATCCAGAAGGCTCAAGTGTCGGGACAAGGTGACAGTCT
GCACGAGCATATCGCCAACCTTGCGGGATCTCCTGCAATCAAGAAGGGTATTCTGCAGACAGTCA
AGGTTGTGGATGAGCTTGTGAAGGTCATGGGACGGCATAAGCCCGAGAACATCGTTATTGAGATG
GCCAGAGAAAATCAGACCACACAAAAGGGTCAGAAGAACTCGAGGGAGCGCATGAAGCGCATCGA
GGAAGGCATTAAGGAGCTGGGGAGTCAGATCCTTAAGGAGCACCCGGTGGAAAACACGCAGTTGC
AAAATGAGAAGCTCTATCTGTACTATCTGCAAAATGGCAGGGATATGTATGTGGACCAGGAGTTG
GATATTAACCGCCTCTCGGATTACGACGTCGATCATATCGTTCCTCAGTCCTTCCTTAAGGATGA
CAGCATTGACAATAAGGTTCTCACCAGGTCCGACAAGAACCGCGGGAAGTCCGATAATGTGCCCA
GCGAGGAAGTCGTTAAGAAGATGAAGAACTACTGGAGGCAACTTTTGAATGCCAAGTTGATCACA
CAGAGGAAGTTTGATAACCTCACTAAGGCCGAGCGCGGAGGTCTCAGCGAACTGGACAAGGCGGG
CTTCATTAAGCGGCAACTGGTTGAGACTAGACAGATCACGAAGCACGTGGCGCAGATTCTCGATT
CACGCATGAACACGAAGTACGATGAGAATGACAAGCTGATCCGGGAAGTGAAGGTCATCACCTTG
AAGTCAAAGCTCGTTTCTGACTTCAGGAAGGATTTCCAATTTTATAAGGTGCGCGAGATCAACAA
TTATCACCATGCTCATGACGCATACCTCAACGCTGTGGTCGGAACAGCATTGATTAAGAAGTACC

CGAAGCTCGAGTCCGAATTCGTGTACGGTGACTATAAGGTTTACGATGTGCGCAAGATGATCGCC
AAGTCAGAGCAGGAAATTGGCAAGGCCACTGCGAAGTATTTCTTTTACTCTAACATTATGAATTT
CTTTAAGACTGAGATCACGCTGGCTAATGGCGAAATCCGGAAGAGACCACTTATTGAGACCAACG
GCGAGACAGGGGAAATCGTGTGGGACAAGGGGAGGGATTTCGCCACAGTCCGCAAGGTTCTCTCT
ATGCCTCAAGTGAATATTGTCAAGAAGACTGAAGTCCAGACGGGCGGGTTCTCAAAGGAATCTAT
TCTGCCCAAGCGGAACTCGGATAAGCTTATCGCCAGAAAGAAGGACTGGGACCCGAAGAAGTATG
GAGGTTTCGACTCACCAACGGTGGCTTACTCTGTCCTGGTTGTGGCAAAGGTGGAGAAGGGAAAG
TCAAAGAAGCTCAAGTCTGTCAAGGAGCTCCTGGGTATCACCATTATGGAGAGGTCCAGCTTCGA
AAAGAATCCGATCGATTTTCTCGAGGCGAAGGGATATAAGGAAGTGAAGAAGGACCTGATCATTA
AGCTTCCAAAGTACAGTCTTTTCGAGTTGGAAAACGGCAGGAAGCGCATGTTGGCTTCCGCAGGA
GAGCTCCAGAAGGGTAACGAGCTTGCTTTGCCGTCCAAGTATGTGAACTTCCTCTATCTGGCATC
CCACTACGAGAAGCTCAAGGGCAGCCCAGAGGATAACGAACAGAAGCAACTGTTTGTGGAGCAAC
ACAAGCATTATCTTGACGAGATCATTGAACAGATTTCGGAGTTCAGTAAGCGCGTCATCCTCGCC
GACGCGAATTTGGATAAGGTTCTCTCAGCCTACAACAAGCACCGGGACAAGCCTATCAGAGAGCA
GGCGGAAAATATCATTCATCTCTTCACCCTGACAAACCTTGGGGCTCCCGCTGCATTCAAGTATT
TTGACACTACGATTGATCGGAAGAGATACACTTCTACGAAGGAGGTGCTGGATGCAACCCTTATC
CACCAATCGATTACTGGCCTCTACGAGACGCGGATCGACTTGAGTCAGCTCGGGGGGGATAAGAG
ACCAGCGGCAACCAAGAAGGCAGGACAAGCGAAGAAGAAGAAGTAG

eSpCas9(1.0)

ATGGCCCCTAAGAAGAAGAGAAAGGTCGGTATTCACGGCGTTCCTGCGGCGATGGACAAGAAGTA
TAGTATTGGTCTGGACATTGGGACGAATTCCGTTGGCTGGGCCGTGATCACCGATGAGTACAAGG
TCCCTTCCAAGAAGTTTAAGGTTCTGGGGAACACCGATCGGCACAGCATCAAGAAGAATCTCATT
GGAGCCCTCCTGTTCGACTCAGGCGAGACCGCCGAAGCAACAAGGCTCAAGAGAACCGCAAGGAG
ACGGTATACAAGAAGGAAGAATAGGATCTGCTACCTGCAGGAGATTTTCAGCAACGAAATGGCGA
AGGTGGACGATTCGTTCTTTCATAGATTGGAGGAGAGTTTCCTCGTCGAGGAAGATAAGAAGCAC
GAGAGGCATCCTATCTTTGGCAACATTGTCGACGAGGTTGCCTATCACGAAAAGTACCCCACAAT
CTATCATCTGCGGAAGAAGCTTGTGGACTCGACTGATAAGGCGGACCTTAGATTGATCTACCTCG
CTCTGGCACACATGATTAAGTTCAGGGGCCATTTTCTGATCGAGGGGGATCTTAACCCGGACAAT
AGCGATGTGGACAAGTTGTTCATCCAGCTCGTCCAAACCTACAATCAGCTCTTTGAGGAAAACCC
AATTAATGCTTCAGGCGTCGACGCCAAGGCGATCCTGTCTGCACGCCTTTCAAAGTCTCGCCGGC
TTGAGAACTTGATCGCTCAACTCCCGGGCGAAAAGAAGAACGGCTTGTTCGGGAATCTCATTGCA
CTTTCGTTGGGGCTCACACCAAACTTCAAGAGTAATTTTGATCTCGCTGAGGACGCAAAGCTGCA
GCTTTCCAAGGACACTTATGACGATGACCTGGATAACCTTTTGGCCCAAATCGGCGATCAGTACG
CGGACTTGTTCCTCGCCGCGAAGAATTTGTCGGACGCGATCCTCCTGAGTGATATTCTCCGCGTG
AACACCGAGATTACAAAGGCCCCGCTCTCGGCGAGTATGATCAAGCGCTATGACGAGCACCATCA
GGATCTGACCCTTTTGAAGGCTTTGGTCCGGCAGCAACTCCCAGAGAAGTACAAGGAAATCTTCT
TTGATCAATCCAAGAACGGCTACGCTGGTTATATTGACGGCGGGGCATCGCAGGAGGAATTCTAC
AAGTTTATCAAGCCAATTCTGGAGAAGATGGATGGCACAGAGGAACTCCTGGTGAAGCTCAATAG
GGAGGACCTTTTGCGGAAGCAAAGAACTTTCGATAACGGCAGCATCCCTCACCAGATTCATCTCG
GGGAGCTGCACGCCATCCTGAGAAGGCAGGAAGACTTCTACCCCTTTCTTAAGGATAACCGGGAG
AAGATCGAAAAGATTCTGACGTTCAGAATTCCGTACTATGTCGGACCACTCGCCCGGGGTAATTC
CAGATTTGCGTGGATGACCAGAAAGAGCGAGGAAACCATCACACCTTGGAACTTCGAGGAAGTGG
TCGATAAGGGCGCTTCCGCACAGAGCTTCATTGAGCGCATGACAAATTTTGACAAGAACCTGCCT
AATGAGAAGGTCCTTCCCAAGCATTCCCTCCTGTACGAGTATTTCACTGTTTATAACGAACTCAC
GAAGGTGAAGTATGTGACCGAGGGAATGCGCAAGCCCGCCTTCCTGAGCGGCGAGCAAAAGAAGG
CGATCGTGGACCTTTTGTTTAAGACCAATCGGAAGGTCACAGTTAAGCAGCTCAAGGAGGACTAC
TTCAAGAAGATTGAATGCTTCGATTCCGTTGAGATCAGCGGCGTGGAAGACAGGTTTAACGCGTC
ACTGGGGACTTACCACGATCTCCTGAAGATCATTAAGGATAAGGACTTCTTGGACAACGAGGAAA
ATGAGGATATCCTCGAAGACATTGTCCTGACTCTTACGTTGTTTGAGGATAGGGAAATGATCGAG
GAACGCTTGAAGACGTATGCCCATCTCTTCGATGACAAGGTTATGAAGCAGCTCAAGAGAAGAAG
ATACACCGGATGGGGAAGGCTGTCCCGCAAGCTTATCAATGGCATTAGAGACAAGCAATCAGGGA
AGACAATCCTTGACTTTTTGAAGTCTGATGGCTTCGCGAACAGGAATTTTATGCAGCTGATTCAC
GATGACTCACTTACTTTCAAGGAGGATATCCAGAAGGCTCAAGTGTCGGGACAAGGTGACAGTCT
GCACGAGCATATCGCCAACCTTGCGGGATCTCCTGCAATCAAGAAGGGTATTCTGCAGACAGTCA
AGGTTGTGGATGAGCTTGTGAAGGTCATGGACGGCATAAGCCCGAGAACATCGTTATTGAGATG
GCCAGAGAAAATCAGACCACACAAAAGGGTCAGAAGAACTCGAGGGAGCGCATGAAGCGCATCGA
GGAAGGCATTAAGGAGCTGGGGAGTCAGATCCTTAAGGAGCACCCGGTGGAAAACACGCAGTTGC

AAAATGAGGCCCTCTATCTGTACTATCTGCAAAATGGCAGGGATATGTATGTGGACCAGGAGTTG
GATATTAACCGCCTCTCGGATTACGACGTCGATCATATCGTTCCTCAGTCCTTCCTTAAGGATGA
CAGCATTGACAATAAGGTTCTCACCAGGTCCGACAAGAACCGCGGGAAGTCCGATAATGTGCCCA
GCGAGGAAGTCGTTAAGAAGATGAAGAACTACTGGAGGCAACTTTTGAATGCCAAGTTGATCACA
CAGAGGAAGTTTGATAACCTCACTAAGGCCGAGCGCGGAGGTCTCAGCGAACTGGACAAGGCGGG
CTTCATTAAGCGGCAACTGGTTGAGACTAGACAGATCACGAAGCACGTGGCGCAGATTCTCGATT
CACGCATGAACACGAAGTACGATGAGAATGACAAGCTGATCCGGGAAGTGAAGGTCATCACCTTG
AAGTCAAAGCTCGTTTCTGACTTCAGGAAGGATTTCCAATTTTATAAGGTGCGCGAGATCAACAA
TTATCACCATGCTCATGACGCATACCTCAACGCTGTGGTCGGAACAGCATTGATTAAGAAGTACC
CGGCGCTCGAGTCCGAATTCGTGTACGGTGACTATAAGGTTTACGATGTGCGCAAGATGATCGCC
AAGTCAGAGCAGGAAATTGGCAAGGCCACTGCGAAGTATTTCTTTTACTCTAACATTATGAATTT
CTTTAAGACTGAGATCACGCTGGCTAATGGCGAAATCCGGAAGGCGCCACTTATTGAGACCAACG
GCGAGACAGGGGAAATCGTGTGGGACAAGGGGAGGGATTTCGCCACAGTCCGCAAGGTTCTCTCT
ATGCCTCAAGTGAATATTGTCAAGAAGACTGAAGTCCAGACGGGCGGGTTCTCAAAGGAATCTAT
TCTGCCCAAGCGGAACTCGGATAAGCTTATCGCCAGAAAGAAGGACTGGGACCCGAAGAAGTATG
GAGGTTTCGACTCACCAACGGTGGCTTACTCTGTCCTGGTTGTGGCAAAGGTGGAGAAGGGAAAG
TCAAAGAAGCTCAAGTCTGTCAAGGAGCTCCTGGGTATCACCATTATGGAGAGGTCCAGCTTCGA
AAAGAATCCGATCGATTTTCTCGAGGCGAAGGGATATAAGGAAGTGAAGAAGGACCTGATCATTA
AGCTTCCAAAGTACAGTCTTTTCGAGTTGGAAAACGGCAGGAAGCGCATGTTGGCTTCCGCAGGA
GAGCTCCAGAAGGGTAACGAGCTTGCTTTGCCGTCCAAGTATGTGAACTTCCTCTATCTGGCATC
CCACTACGAGAAGCTCAAGGGCAGCCCAGAGGATAACGAACAGAAGCAACTGTTTGTGGAGCAAC
ACAAGCATTATCTTGACGAGATCATTGAACAGATTTCGGAGTTCAGTAAGCGCGTCATCCTCGCC
GACGCGAATTTGGATAAGGTTCTCTCAGCCTACAACAAGCACCGGGACAAGCCTATCAGAGAGCA
GGCGGAAAATATCATTCATCTCTTCACCCTGACAAACCTTGGGGCTCCCGCTGCATTCAAGTATT
TTGACACTACGATTGATCGGAAGAGATACACTTCTACGAAGGAGGTGCTGGATGCAACCCTTATC
CACCAATCGATTACTGGCCTCTACGAGACGCGGATCGACTTGAGTCAGCTCGGGGGGGATAAGAG
ACCAGCGGCAACCAAGAAGGCAGGACAAGCGAAGAAGAAGAAGTAG

eSpCas9(1.1)

ATGGCCCCTAAGAAGAAGAGAAAGGTCGGTATTCACGGCGTTCCTGCGGCGATGGACAAGAAGTA
TAGTATTGGTCTGGACATTGGGACGAATTCCGTTGGCTGGGCCGTGATCACCGATGAGTACAAGG
TCCCTTCCAAGAAGTTTAAGGTTCTGGGGAACACCGATCGGCACAGCATCAAGAAGAATCTCATT
GGAGCCCTCCTGTTCGACTCAGGCGAGACCGCCGAAGCAACAAGGCTCAAGAGAACCGCAAGGAG
ACGGTATACAAGAAGGAAGAATAGGATCTGCTACCTGCAGGAGATTTTCAGCAACGAAATGGCGA
AGGTGGACGATTCGTTCTTTCATAGATTGGAGGAGAGTTTCCTCGTCGAGGAAGATAAGAAGCAC
GAGAGGCATCCTATCTTTGGCAACATTGTCGACGAGGTTGCCTATCACGAAAAGTACCCCACAAT
CTATCATCTGCGGAAGAAGCTTGTGGACTCGACTGATAAGGCGGACCTTAGATTGATCTACCTCG
CTCTGGCACACATGATTAAGTTCAGGGGCCATTTTCTGATCGAGGGGGATCTTAACCCGGACAAT
AGCGATGTGGACAAGTTGTTCATCCAGCTCGTCCAAACCTACAATCAGCTCTTTGAGGAAAACCC
AATTAATGCTTCAGGCGTCGACGCCAAGGCGATCCTGTCTGCACGCCTTTCAAAGTCTCGCCGGC
TTGAGAACTTGATCGCTCAACTCCCGGGCGAAAAGAAGAACGGCTTGTTCGGGAATCTCATTGCA
CTTTCGTTGGGGCTCACACCAAACTTCAAGAGTAATTTTGATCTCGCTGAGGACGCAAAGCTGCA
GCTTTCCAAGGACACTTATGACGATGACCTGGATAACCTTTTGGCCCAAATCGGCGATCAGTACG
CGGACTTGTTCCTCGCCGCGAAGAATTTGTCGGACGCGATCCTCCTGAGTGATATTCTCCGCGTG
AACACCGAGATTACAAAGGCCCCGCTCTCGGCGAGTATGATCAAGCGCTATGACGAGCACCATCA
GGATCTGACCCTTTTGAAGGCTTTGGTCCGGCAGCAACTCCCAGAGAAGTACAAGGAAATCTTCT
TTGATCAATCCAAGAACGGCTACGCTGGTTATATTGACGGCGGGGCATCGCAGGAGGAATTCTAC
AAGTTTATCAAGCCAATTCTGGAGAAGATGGATGGCACAGAGGAACTCCTGGTGAAGCTCAATAG
GGAGGACCTTTTGCGGAAGCAAAGAACTTTCGATAACGGCAGCATCCCTCACCAGATTCATCTCG
GGGAGCTGCACGCCATCCTGAGAAGGCAGGAAGACTTCTACCCCTTTCTTAAGGATAACCGGGAG
AAGATCGAAAAGATTCTGACGTTCAGAATTCCGTACTATGTCGGACCACTCGCCCGGGGTAATTC
CAGATTTGCGTGGATGACCAGAAAGAGCGAGGAAACCATCACACCTTGGAACTTCGAGGAAGTGG
TCGATAAGGGCGCTTCCGCACAGAGCTTCATTGAGCGCATGACAAATTTTGACAAGAACCTGCCT
AATGAGAAGGTCCTTCCCAAGCATTCCCTCCTGTACGAGTATTTCACTGTTTATAACGAACTCAC
GAAGGTGAAGTATGTGACCGAGGGAATGCGCAAGCCCGCCTTCCTGAGCGGCGAGCAAAAGAAGG
CGATCGTGGACCTTTTGTTTAAGACCAATCGGAAGGTCACAGTTAAGCAGCTCAAGGAGGACTAC
TTCAAGAAGATTGAATGCTTCGATTCCGTTGAGATCAGCGGCGTGGAAGACAGGTTTAACGCGTC
ACTGGGGACTTACCACGATCTCCTGAAGATCATTAAGGATAAGGACTTCTTGGACAACGAGGAAA

ATGAGGATATCCTCGAAGACATTGTCCTGACTCTTACGTTGTTTGAGGATAGGGAAATGATCGAG
GAACGCTTGAAGACGTATGCCCATCTCTTCGATGACAAGGTTATGAAGCAGCTCAAGAGAAGAAG
ATACACCGGATGGGGAAGGCTGTCCCGCAAGCTTATCAATGGCATTAGAGACAAGCAATCAGGGA
AGACAATCCTTGACTTTTTGAAGTCTGATGGCTTCGCGAACAGGAATTTTATGCAGCTGATTCAC
GATGACTCACTTACTTTCAAGGAGGATATCCAGAAGGCTCAAGTGTCGGGACAAGGTGACAGTCT
GCACGAGCATATCGCCAACCTTGCGGGATCTCCTGCAATCAAGAAGGGTATTCTGCAGACAGTCA
AGGTTGTGGATGAGCTTGTGAAGGTCATGGGACGGCATAAGCCCGAGAACATCGTTATTGAGATG
GCCAGAGAAAATCAGACCACACAAAAGGGTCAGAAGAACTCGAGGGAGCGCATGAAGCGCATCGA
GGAAGGCATTAAGGAGCTGGGGAGTCAGATCCTTAAGGAGCACCCGGTGGAAAACACGCAGTTGC
AAAATGAGAAGCTCTATCTGTACTATCTGCAAAATGGCAGGGATATGTATGTGGACCAGGAGTTG
GATATTAACCGCCTCTCGGATTACGACGTCGATCATATCGTTCCTCAGTCCTTCCTTGCGGATGA
CAGCATTGACAATAAGGTTCTCACCAGGTCCGACAAGAACCGCGGGAAGTCCGATAATGTGCCCA
GCGAGGAAGTCGTTAAGAAGATGAAGAACTACTGGAGGCAACTTTTGAATGCCAAGTTGATCACA
CAGAGGAAGTTTGATAACCTCACTAAGGCCGAGCGCGGAGGTCTCAGCGAACTGGACAAGGCGGG
CTTCATTAAGCGGCAACTGGTTGAGACTAGACAGATCACGAAGCACGTGGCGCAGATTCTCGATT
CACGCATGAACACGAAGTACGATGAGAATGACAAGCTGATCCGGGAAGTGAAGGTCATCACCTTG
AAGTCAAAGCTCGTTTCTGACTTCAGGAAGGATTTCCAATTTTATAAGGTGCGCGAGATCAACAA
TTATCACCATGCTCATGACGCATACCTCAACGCTGTGGTCGGAACAGCATTGATTAAGAAGTACC
CGGCGCTCGAGTCCGAATTCGTGTACGGTGACTATAAGGTTTACGATGTGCGCAAGATGATCGCC
AAGTCAGAGCAGGAAATTGGCAAGGCCACTGCGAAGTATTTCTTTTACTCTAACATTATGAATTT
CTTTAAGACTGAGATCACGCTGGCTAATGGCGAAATCCGGAAGGCGCCACTTATTGAGACCAACG
GCGAGACAGGGGAAATCGTGTGGGACAAGGGGAGGGATTTCGCCACAGTCCGCAAGGTTCTCTCT
ATGCCTCAAGTGAATATTGTCAAGAAGACTGAAGTCCAGACGGGCGGGTTCTCAAAGGAATCTAT
TCTGCCCAAGCGGAACTCGGATAAGCTTATCGCCAGAAAGAAGGACTGGGACCCGAAGAAGTATG
GAGGTTTCGACTCACCAACGGTGGCTTACTCTGTCCTGGTTGTGGCAAAGGTGGAGAAGGGAAAG
TCAAAGAAGCTCAAGTCTGTCAAGGAGCTCCTGGGTATCACCATTATGGAGAGGTCCAGCTTCGA
AAAGAATCCGATCGATTTTCTCGAGGCGAAGGGATATAAGGAAGTGAAGAAGGACCTGATCATTA
AGCTTCCAAAGTACAGTCTTTTCGAGTTGGAAAACGGCAGGAAGCGCATGTTGGCTTCCGCAGGA
GAGCTCCAGAAGGGTAACGAGCTTGCTTTGCCGTCCAAGTATGTGAACTTCCTCTATCTGGCATC
CCACTACGAGAAGCTCAAGGGCAGCCCAGAGGATAACGAACAGAAGCAACTGTTTGTGGAGCAAC
ACAAGCATTATCTTGACGAGATCATTGAACAGATTTCGGAGTTCAGTAAGCGCGTCATCCTCGCC
GACGCGAATTTGGATAAGGTTCTCTCAGCCTACAACAAGCACCGGGACAAGCCTATCAGAGAGCA
GGCGGAAAATATCATTCATCTCTTCACCCTGACAAACCTTGGGGCTCCCGCTGCATTCAAGTATT
TTGACACTACGATTGATCGGAAGAGATACACTTCTACGAAGGAGGTGCTGGATGCAACCCTTATC
CACCAATCGATTACTGGCCTCTACGAGACGCGGATCGACTTGAGTCAGCTCGGGGGGGATAAGAG
ACCAGCGGCAACCAAGAAGGCAGGACAAGCGAAGAAGAAGAAGTAG

SpCas9-HF1

ATGGCCCCTAAGAAGAAGAGAAAGGTCGGTATTCACGGCGTTCCTGCGGCGATGGACAAGAAGTA
TAGTATTGGTCTGGACATTGGGACGAATTCCGTTGGCTGGGCCGTGATCACCGATGAGTACAAGG
TCCCTTCCAAGAAGTTTAAGGTTCTGGGGAACACCGATCGGCACAGCATCAAGAAGAATCTCATT
GGAGCCCTCCTGTTCGACTCAGGCGAGACCGCCGAAGCAACAAGGCTCAAGAGAACCGCAAGGAG
ACGGTATACAAGAAGGAAGAATAGGATCTGCTACCTGCAGGAGATTTTCAGCAACGAAATGGCGA
AGGTGGACGATTCGTTCTTTCATAGATTGGAGGAGAGTTTCCTCGTCGAGGAAGATAAGAAGCAC
GAGAGGCATCCTATCTTTGGCAACATTGTCGACGAGGTTGCCTATCACGAAAAGTACCCCACAAT
CTATCATCTGCGGAAGAAGCTTGTGGACTCGACTGATAAGGCGGACCTTAGATTGATCTACCTCG
CTCTGGCACACATGATTAAGTTCAGGGGCCATTTTCTGATCGAGGGGGATCTTAACCCGGACAAT
AGCGATGTGGACAAGTTGTTCATCCAGCTCGTCCAAACCTACAATCAGCTCTTTGAGGAAAACCC
AATTAATGCTTCAGGCGTCGACGCCAAGGCGATCCTGTCTGCACGCCTTTCAAAGTCTCGCCGGC
TTGAGAACTTGATCGCTCAACTCCCGGGCGAAAAGAAGAACGGCTTGTTCGGGAATCTCATTGCA
CTTTCGTTGGGGCTCACACCAAACTTCAAGAGTAATTTTGATCTCGCTGAGGACGCAAAGCTGCA
GCTTTCCAAGGACACTTATGACGATGACCTGGATAACCTTTTGGCCCAAATCGGCGATCAGTACG
CGGACTTGTTCCTCGCCGCGAAGAATTTGTCGGACGCGATCCTCCTGAGTGATATTCTCCGCGTG
AACACCGAGATTACAAAGGCCCCGCTCTCGGCGAGTATGATCAAGCGCTATGACGAGCACCATCA
GGATCTGACCCTTTTGAAGGCTTTGGTCCGGCAGCAACTCCCAGAGAAGTACAAGGAAATCTTCT
TTGATCAATCCAAGAACGGCTACGCTGGTTATATTGACGGCGGGGCATCGCAGGAGGAATTCTAC
AAGTTTATCAAGCCAATTCTGGAGAAGATGGATGGCACAGAGGAACTCCTGGTGAAGCTCAATAG
GGAGGACCTTTTGCGGAAGCAAAGAACTTTCGATAACGGCAGCATCCCTCACCAGATTCATCTCG

GGGAGCTGCACGCCATCCTGAGAAGGCAGGAAGACTTCTACCCCTTTCTTAAGGATAACCGGGAG
AAGATCGAAAAGATTCTGACGTTCAGAATTCCGTACTATGTCGGACCACTCGCCCGGGGTAATTC
CAGATTTGCGTGGATGACCAGAAAGAGCGAGGAAACCATCACACCTTGGAACTTCGAGGAAGTGG
TCGATAAGGGCGCTTCCGCACAGAGCTTCATTGAGCGCATGACAGCCTTTGACAAGAACCTGCCT
AATGAGAAGGTCCTTCCCAAGCATTCCCTCCTGTACGAGTATTTCACTGTTTATAACGAACTCAC
GAAGGTGAAGTATGTGACCGAGGGAATGCGCAAGCCCGCCTTCCTGAGCGGCGAGCAAAAGAAGG
CGATCGTGGACCTTTTGTTTAAGACCAATCGGAAGGTCACAGTTAAGCAGCTCAAGGAGGACTAC
TTCAAGAAGATTGAATGCTTCGATTCCGTTGAGATCAGCGGCGTGGAAGACAGGTTTAACGCGTC
ACTGGGGACTTACCACGATCTCCTGAAGATCATTAAGGATAAGGACTTCTTGGACAACGAGGAAA
ATGAGGATATCCTCGAAGACATTGTCCTGACTCTTACGTTGTTTGAGGATAGGGAAATGATCGAG
GAACGCTTGAAGACGTATGCCCATCTCTTCGATGACAAGGTTATGAAGCAGCTCAAGAGAAGAAG
ATACACCGGATGGGGAGCCCTGTCCCGCAAGCTTATCAATGGCATTAGAGACAAGCAATCAGGGA
AGACAATCCTTGACTTTTTGAAGTCTGATGGCTTCGCGAACAGGAATTTTATGGCCCTGATTCAC
GATGACTCACTTACTTTCAAGGAGGATATCCAGAAGGCTCAAGTGTCGGGACAAGGTGACAGTCT
GCACGAGCATATCGCCAACCTTGCGGGATCTCCTGCAATCAAGAAGGGTATTCTGCAGACAGTCA
AGGTTGTGGATGAGCTTGTGAAGGTCATGGGACGGCATAAGCCCGAGAACATCGTTATTGAGATG
GCCAGAGAAAATCAGACCACACAAAAGGGTCAGAAGAACTCGAGGGAGCGCATGAAGCGCATCGA
GGAAGGCATTAAGGAGCTGGGGAGTCAGATCCTTAAGGAGCACCCGGTGGAAAACACGCAGTTGC
AAAATGAGAAGCTCTATCTGTACTATCTGCAAAATGGCAGGGATATGTATGTGGACCAGGAGTTG
GATATTAACCGCCTCTCGGATTACGACGTCGATCATATCGTTCCTCAGTCCTTCCTTAAGGATGA
CAGCATTGACAATAAGGTTCTCACCAGGTCCGACAAGAACCGCGGGAAGTCCGATAATGTGCCCA
GCGAGGAAGTCGTTAAGAAGATGAAGAACTACTGGAGGCAACTTTTGAATGCCAAGTTGATCACA
CAGAGGAAGTTTGATAACCTCACTAAGGCCGAGCGCGGAGGTCTCAGCGAACTGGACAAGGCGGG
CTTCATTAAGCGGCAACTGGTTGAGACTAGAGCCATCACGAAGCACGTGGCGCAGATTCTCGATT
CACGCATGAACACGAAGTACGATGAGAATGACAAGCTGATCCGGGAAGTGAAGGTCATCACCTTG
AAGTCAAAGCTCGTTTCTGACTTCAGGAAGGATTTCCAATTTTATAAGGTGCGCGAGATCAACAA
TTATCACCATGCTCATGACGCATACCTCAACGCTGTGGTCGGAACAGCATTGATTAAGAAGTACC
CGAAGCTCGAGTCCGAATTCGTGTACGGTGACTATAAGGTTTACGATGTGCGCAAGATGATCGCC
AAGTCAGAGCAGGAAATTGGCAAGGCCACTGCGAAGTATTTCTTTTACTCTAACATTATGAATTT
CTTTAAGACTGAGATCACGCTGGCTAATGGCGAAATCCGGAAGAGACCACTTATTGAGACCAACG
GCGAGACAGGGGAAATCGTGTGGGACAAGGGGAGGGATTTCGCCACAGTCCGCAAGGTTCTCTCT
ATGCCTCAAGTGAATATTGTCAAGAAGACTGAAGTCCAGACGGGCGGGTTCTCAAAGGAATCTAT
TCTGCCCAAGCGGAACTCGGATAAGCTTATCGCCAGAAAGAAGGACTGGGACCCGAAGAAGTATG
GAGGTTTCGACTCACCAACGGTGGCTTACTCTGTCCTGGTTGTGGCAAAGGTGGAGAAGGGAAAG
TCAAAGAAGCTCAAGTCTGTCAAGGAGCTCCTGGGTATCACCATTATGGAGAGGTCCAGCTTCGA
AAAGAATCCGATCGATTTTCTCGAGGCGAAGGGATATAAGGAAGTGAAGAAGGACCTGATCATTA
AGCTTCCAAAGTACAGTCTTTTCGAGTTGGAAAACGGCAGGAAGCGCATGTTGGCTTCCGCAGGA
GAGCTCCAGAAGGGTAACGAGCTTGCTTTGCCGTCCAAGTATGTGAACTTCCTCTATCTGGCATC
CCACTACGAGAAGCTCAAGGGCAGCCCAGAGGATAACGAACAGAAGCAACTGTTTGTGGAGCAAC
ACAAGCATTATCTTGACGAGATCATTGAACAGATTTCGGAGTTCAGTAAGCGCGTCATCCTCGCC
GACGCGAATTTGGATAAGGTTCTCTCAGCCTACAACAAGCACCGGGACAAGCCTATCAGAGAGCA
GGCGGAAAATATCATTCATCTCTTCACCCTGACAAACCTTGGGGCTCCCGCTGCATTCAAGTATT
TTGACACTACGATTGATCGGAAGAGATACACTTCTACGAAGGAGGTGCTGGATGCAACCCTTATC
CACCAATCGATTACTGGCCTCTACGAGACGCGGATCGACTTGAGTCAGCTCGGGGGGGATAAGAG
ACCAGCGGCAACCAAGAAGGCAGGACAAGCGAAGAAGAAGAAGTAG
